# Quaternary Structure Transitions of Human Hemoglobin: An Atomic-Level View of the Functional Intermediate States

**DOI:** 10.1021/acs.jcim.1c00315

**Published:** 2021-08-10

**Authors:** Nicole Balasco, Josephine Alba, Marco D’Abramo, Luigi Vitagliano

**Affiliations:** † Institute of Biostructures and Bioimaging, CNR, Via Mezzocannone 16, 80134 Naples, Italy; ‡ Department of Chemistry, University of Rome Sapienza, P.le A.Moro 5, 00185 Rome, Italy

## Abstract

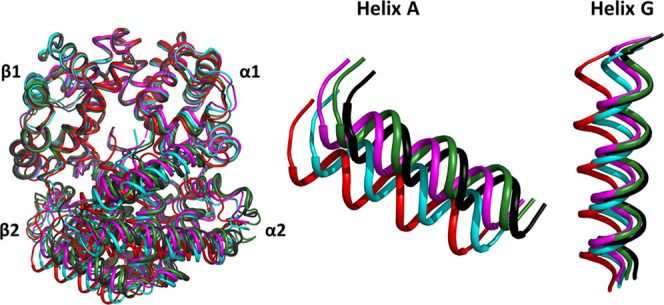

Human hemoglobin (HbA) is one of the prototypal systems used to investigate structure–function relationships in proteins. Indeed, HbA has been used to develop the basic concepts of protein allostery, although the atomic-level mechanism underlying the HbA functionality is still highly debated. This is due to the fact that most of the three-dimensional structural information collected over the decades refers to the endpoints of HbA functional transition with little data available for the intermediate states. Here, we report molecular dynamics (MD) simulations by focusing on the relevance of the intermediate states of the protein functional transition unraveled by the crystallographic studies carried out on vertebrate Hbs. Fully atomistic simulations of the HbA T-state indicate that the protein undergoes a spontaneous transition toward the R-state. The inspection of the trajectory structures indicates that the protein significantly populates the intermediate HL-(C) state previously unraveled by crystallography. In the structural transition, it also assumes the intermediate states crystallographically detected in Antarctic fish Hbs. This finding suggests that HbA and Antarctic fish Hbs, in addition to the endpoints of the transitions, also share a similar deoxygenation pathway despite a distace of hundreds of millions of years in the evolution scale. Finally, using the essential dynamic sampling methodology, we gained some insights into the reverse R to T transition that is not spontaneously observed in classic MD simulations.

## Introduction

Human hemoglobin (HbA) deserves a special position in structural biology since it has been the model used to develop the fundaments of protein crystallography.^1−5^ Moreover, myoglobin has been the first protein whose structure has been determined at an atomic level.^6^ Even more significantly, HbA has been, and it still is, the prototypal system used to investigate structure–function relationships in proteins. Indeed, the structural characterization of the different functional states of HbA has provided fundamental insights for developing the basic concepts of protein allostery, although the atomic-level mechanism underlying the HbA functionality is still highly debated.^1,5,7−10^ Due to the seminal work of Perutz, the structural features of the endpoints of Hb structural transition have been elucidated for more than half a century.^11^ These pioneering studies have unveiled that the ligand-bound forms are associated with a rather flexible structure denoted as the R (relaxed) state. On the other hand, the unliganded HbA structure is characterized by a rather rigid, tense, T-state.

Since then, hundreds of HbA structures have been reported in the Protein Data Bank (PDB).^7,12^ These studies have significantly improved the structural sampling of the HbA bound states by showing that the liganded HbA may manifest, in addition to the R-state, in a variety of other relaxed states (R2, R3, RR2) that fall outside the T–R pathway (Table S1).^7^ These findings have initiated an intense debate on the real extension of the Hb functional transition that may go well beyond the T–R pathway and include these other relaxed states.^7,13^ On the other hand, the atomic-level characterizations of intermediate R–T states have proven much more difficult as HbA structures exhibiting intermediate R–T features at the quaternary structure level have been rarely described. The first example has been reported by Schumacher et al.,^14^ who generated intermediate R–T states through the crosslinking of the β chains of the HbA tetramer. Interesting information on HbA function–structure relationships were also provided by crystallographic structures showing remarkable tertiary structure variations, although confined either to the R- or the T-state.^7^ More recently, a unique crystal form of HbA characterized by the presence at the crystalline state of three different quaternary structures has been reported.^15,16^ One of these states, denoted as HL-(C), presents intriguing intermediate features, although it is much closer to the R-state rather than to the T-state (Table S1). In this scenario, although insightful information on the R–T transition has been obtained using other experimental and computational techniques,^7,16^ the lack of detailed experimental information on intermediate T–R states is one of the most important factors that has so far prevented a full understanding of the HbA functional transition.

**Figure 1:**
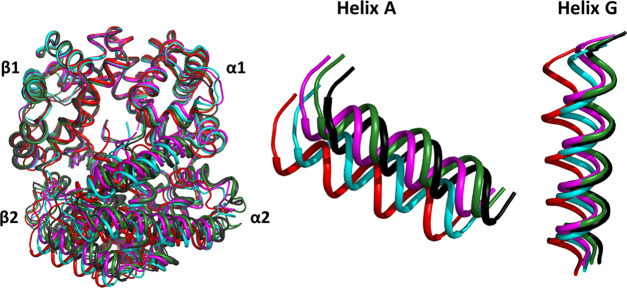
Stepwise R–T transition of HbTn as highlighted by crystallographic investigations carried out on the protein. Superimposition of the α1β1 dimer of different HbTn structural states: deoxygenated T-state (TnT, black, PDB ID: 3NFE), TnA (green, PDB ID: 5LFG), TnB (magenta, PDB ID: 5LFG), TnH (cyan, PDB ID: 3D1K), and the canonical R-state (TnR, red, PDB ID: 1T1N). Magnifications of the helices A (residue 3–18) and G (residues 99–117) of the β2 subunit are shown to highlight the transition from the T- to the R-state.

The situation is significantly different for the tetrameric Hbs isolated from Antarctic fish (AntHbs) that share with HbA several functional/structural features despite the fact that AntHbs operates in organisms living in rather extreme conditions.^17−25^ The intrinsic flexibility of AntHbs when studied at temperatures significantly higher than the physiological ones has allowed the visualization of states that exhibit unusual structural properties and/or peculiar oxidation states. Specifically, the structures of Hbs extracted from *Trematomus newnesi* and *Trematomus bernacchii* belonging to the Nototheniidae family and from the sub-Antarctic fish *Eleginops maclovinus* have shown a variety of oxidation (hemichrome, aquomet, pentacoordinated oxidized, etc.) often associated with noncanonical tertiary and quaternary organizations.^23,26−29^ Altogether, these structures have provided some interesting insights into the possible structural features of the intermediate states (see for example ref (30)). Interestingly, for the *T. newnesi* Hb (HbTn), three distinct intermediate structures (tetramer A, TnA; tetramer B, TnB; and tetramer H, TnH),^26,30^ in addition to the canonical T and R states, have been reported (Figure 1 and Table S1). For HbTn, the overall rotation of one αβ dimer when the other is superimposed is about 11° (15° in the case of HbA). Therefore, taking into account the presence of the three intermediate states, the overall pathway may be dissected into four subtransitions that are separated by approximately 3° rotation of one αβ dimer with respect to the other (Figure 1). In this scenario, to gain further insights into this long-standing issue, we here performed extensive fully atomistic molecular dynamics (MD) simulations on HbA by also checking the relevance of the intermediate species identified for AntHbs to the human counterpart.

## Results

### MD Simulations of the HbA T-State: A Global Analysis of the Trajectory Structures

**Figure 2:**
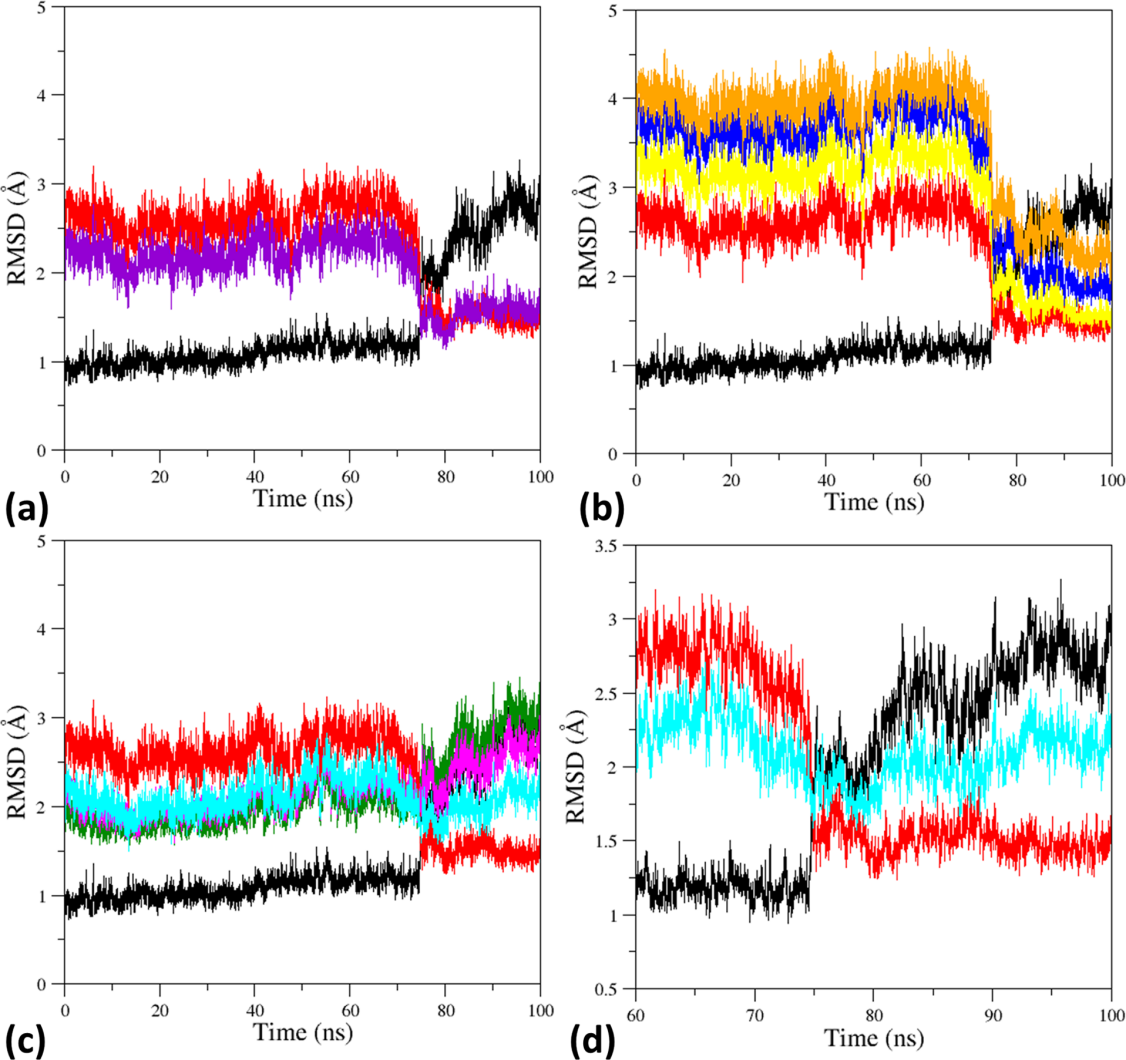
(a) RMSD values (computed on the C^α^ atoms) of the T0 trajectory structures versus the starting T model (black, PDB ID: 2DN2), the R-state (red, PDB ID: 2DN1), and the intermediate HL-(C) state (violet, PDB ID: 4N7P). (b) RMSD values computed against the off-pathway structures: R2 (blue, PDB ID: 1BBB), RR2 (yellow, PDB ID: 1MKO), and R3 (orange, PDB ID: 4NI0). (c) RMSD values computed against the intermediate states identified for HbTn: TnA (green, PDB ID: 5LFG), TnB (magenta, PDB ID: 5LFG), and TnH (cyan, PDB ID: 3D1K) (C). (d) RMSD values computed against the T-state (black) and the R-state (red) of HbA and against TnH (cyan) in the time interval of 60–100 ns. RMSD values refer to the productive run without the equilibration steps producing the initial drift.

The T–R transition was investigated by MD simulations carried out using the fully unligated T-state of HbA as the starting model (T0 simulation). Taking into account the indications that emerged in previous analyses,^8,31^ the terminal His (His146) of the β chains was kept uncharged to favor the transition toward the R-state. Indeed, the electrostatic interaction between the side chain of the terminal His146β and Asp94β is important for the stabilization of the T-state.^11^ As shown in Figure 2, a clear transition is observed within the first 100 ns. Indeed, the analysis of the root-mean-square deviation (RMSD) values of the MD structures against either the R- or the T-state indicates that they suddenly become closer to the R-state while diverging from the T-state (Figure 2a). To gain further insights into the evolution of HbA throughout the simulation, we computed the RMSD values of the trajectory structures with respect to additional, well-characterized, structural states of the protein. Indeed, the RMSD values calculated against the HL-(C) state^14^ follow the trend observed for those computed against the R-state (Figure 2a). It is worth mentioning, however, that in the initial stage of the trajectory, the RMSD values obtained versus the HL-(C) state are significantly lower than those obtained versus the R-state, as expected for this R-like HbA intermediate state. A closer inspection of Figure 2a also indicates that the RMSD values computed against the HL-(C) state present a local minimum at ∼80 ns, when the trajectory structures have almost completed their evolution toward the R-state. Once again, these observations indicate that the HL-(C) state is a real global intermediate state somehow close, however, to the R-state. Then, we evaluated the similarity/dissimilarity of the trajectory structures with respect to the HbA states that are off from the T–R pathway (i.e., R2, RR2, and R3 states). As shown in Figure 2b, the starting RMSD values calculated against these structures are larger than those detected against the R-state. This observation is not surprising considering the position of R2, RR2, and R3 in the T–R pathway. Moreover, since these states are closer to the R-state rather than the T-state, these RMSD values also decrease upon the transition observed in the MD simulation. Notably, after the transition, the RMSD values versus the RR2 state are similar to those displayed versus the R-state. This observation indicates that at the end of the MD transition, the conformational ensemble of the trajectory structures generated by the simulation, although R-like, presents some features of the RR2 state. Similar transitions have been observed in three other independent MD simulation runs (T0b, T0c, and T0d) conducted on the T-state of HbA (Figures S1–S3).

We also compared the T0 trajectory structures with the intermediate states detected in the crystallographic characterizations of *T. newnesi* Hb. Despite the differences in the sequences between HbA and HbTn, similar trends of the RMSD values are observed (Figure 2c). The evolution of the RMSD values computed against these intermediates shows remarkable variations in the correspondence of the T to R transition of HbA that occurs at ∼ 75 ns. The starting values of the RMSD observed for the tetramers A, B, and H of HbTn are intermediate between those observed for the T- and the R-state of HbA. For the tetramers A and B, which display some similarity with the T-state, the RMSD values increase upon the transition in the last part of the trajectory where MD structures assume R-like states. A deep inspection of Figure 2c also indicates that the trajectory structures closest to A and B tetramers are located in the initial stage of the simulations. The observation suggests that these tetramers fall in the conformational basin accessible to the T-state.

For the tetramer H, which is rather distinct from both the T and the R-state, we observe that trajectory structures present the lowest RMSD values in the time interval that corresponds to the T–R transition (Figure 2d).

### MD Simulations of the HbA T-State: Essential Dynamics

**Figure 3:**
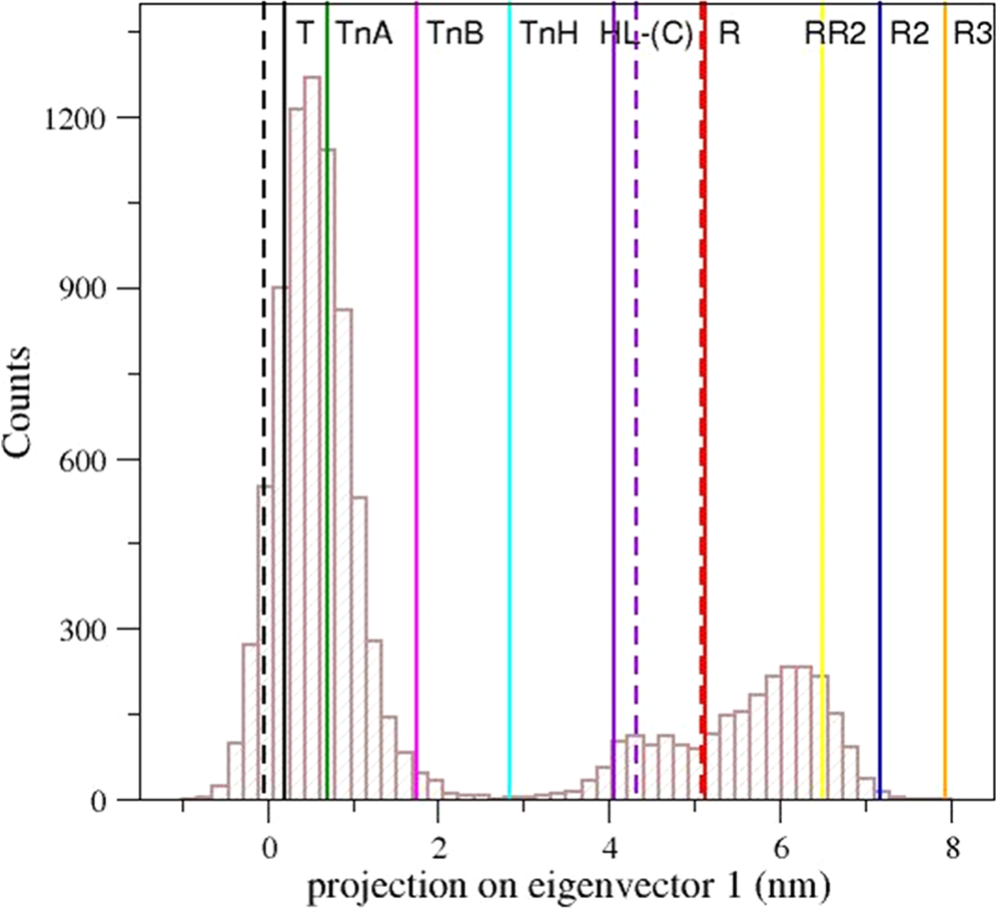
Projection on the first eigenvector of the T0 trajectory structures. The vertical solid lines correspond to the projections of the crystallographic structures of human HbA states: T (black, PDB ID: 2DN2), R (red, PDB ID: 2DN1), intermediate HL-(C) (violet, PDB ID: 4N7P), R2 (blue, PDB ID: 1BBB), RR2 (yellow, PDB ID: 1MKO), and R3 (orange, PDB ID: 4NI0) and of the HbTn intermediates: TnA (green, PDB ID: 5LFG), TnB (magenta, PDB ID: 5LFG), and TnH (cyan, PDB ID: 3D1K). Dashed lines correspond to the projections of crystallographic structures showing remarkable tertiary structure variations as oxy HbA T-state (black, PDB ID: 1GZX) and high-salt carbonmonoxy HbA (red, PDB ID: 1LJW) and of the crystallographic structures of cross-linked carbonmonoxy HbAs (violet, PDB IDs: 1SDK and 1SDL).

To gain further insights into the T–R transition detected in the T0 simulation, the trajectory was also analyzed by means of the essential dynamics (ED) method, in which the principal motion directions of large systems are represented by a set of eigenvectors (see Materials and Methods for further details). The eigenvectors were calculated from the entire ensemble of the trajectory frames obtained from the T0 simulation, and ranked according to their eigenvalues (Figure S4). The structures derived from this simulation and some representative crystallographic models have been projected along with the first principal component (eigenvector) that accounts for ∼60% of the whole protein fluctuation (Figure 3). In line with the expectations, the R2, RR2, and R3 crystallographic structures of HbA are distributed outside of the T–R pathway. In line with the hypothesis that the HbA functional transition may also include these states, structures that emerged from the T0 MD simulation frequently go beyond the R-state.

In this representation, some of the Hb structures showing remarkable tertiary structure variations are close to either the R- or the T-state. On the other hand, the HbA intermediate HL-(C) is located along the pathway, although closer to the R- than to the T-state. In this projection, the cross-linked intermediates identified by Schumacher et al.^14^ lie very close to the HL-(C) state. Interestingly, all of the intermediate states identified in the crystallographic structures of HbTn also fall in the T–R pathway. These observations clearly suggest that the principal motion direction, represented by the first eigenvector, well reproduces the T–R functional transitions. A closer inspection of the distribution also demonstrates that the T0 trajectory structures well populate the states corresponding to the intermediates HL-(C), TnA, and TnB that are not very far from the canonical T or R states. On the other hand, the state TnH, which lies in the middle of the transition, is very poorly populated. The TnH state corresponds to the maximum of the free energy along the trajectory as highlighted by the free energy difference between the sampled states along with the T–R transition that was calculated as the ratio between the H state population and a reference (most-populated) state, Δ*G* = −*RT* ln (*p*_TnH_/*p*_ref_) (Figure S6). This observation is not surprising as HbA undergoes a very sudden T–R transition and, also, might explain why intermediate states for HbA have been so elusive despite the large amount of structural data collected for this protein.

Similar results, in terms of the population of the states lying within the transition, of the structural versatility of the R-state and of the prevalent role played by the first eigenvector have been obtained in the independent T0b, T0c, and T0d MD simulation runs (Figures S1–S6).

### Evolution of the Structural Probes that Characterize the Endpoints of the T–R Transition in the MD Simulation

The analysis of the trajectory structures performed at the global level in the previous section, i.e., by principal component analysis, was extended considering specific structural probes that undergo large variations upon the transition. To this end, suitable structural probes have been initially identified. Then, in the subspace defined by these probes, we located representative crystallographic structures of both HbA and HbTn and then the T0 trajectory structures.

Following the vast HbA literature data and the indications that emerged from a comparative analysis of the T- and the R-state of the protein, we identified local descriptors that assume specific distinct values for each structural state of HbA (Tables S2 and S3). These include (a) the distance between groups/atoms whose interactions specifically stabilize the T-state (K40α1 side chain–H146β2 COOH terminal group and Y42α1–D99β2 side chains), (b) the distance between the residues located in the switch region α1CD−β2FG at the interface between the dimers α1β1 and α2β2 (C^α^ Pro44α1–C^α^ His97β2),^32^ (c) the distance between the C^α^ atoms of the terminal His residues (His146) of the two β chains (C^α^ His146β1–C^α^ His146β2), and (d) some structural features of the heme pocket (C^α^–C^α^ distance between the proximal and distal His residues and the rotameric state of the distal His side chain).

**Figure 4:**
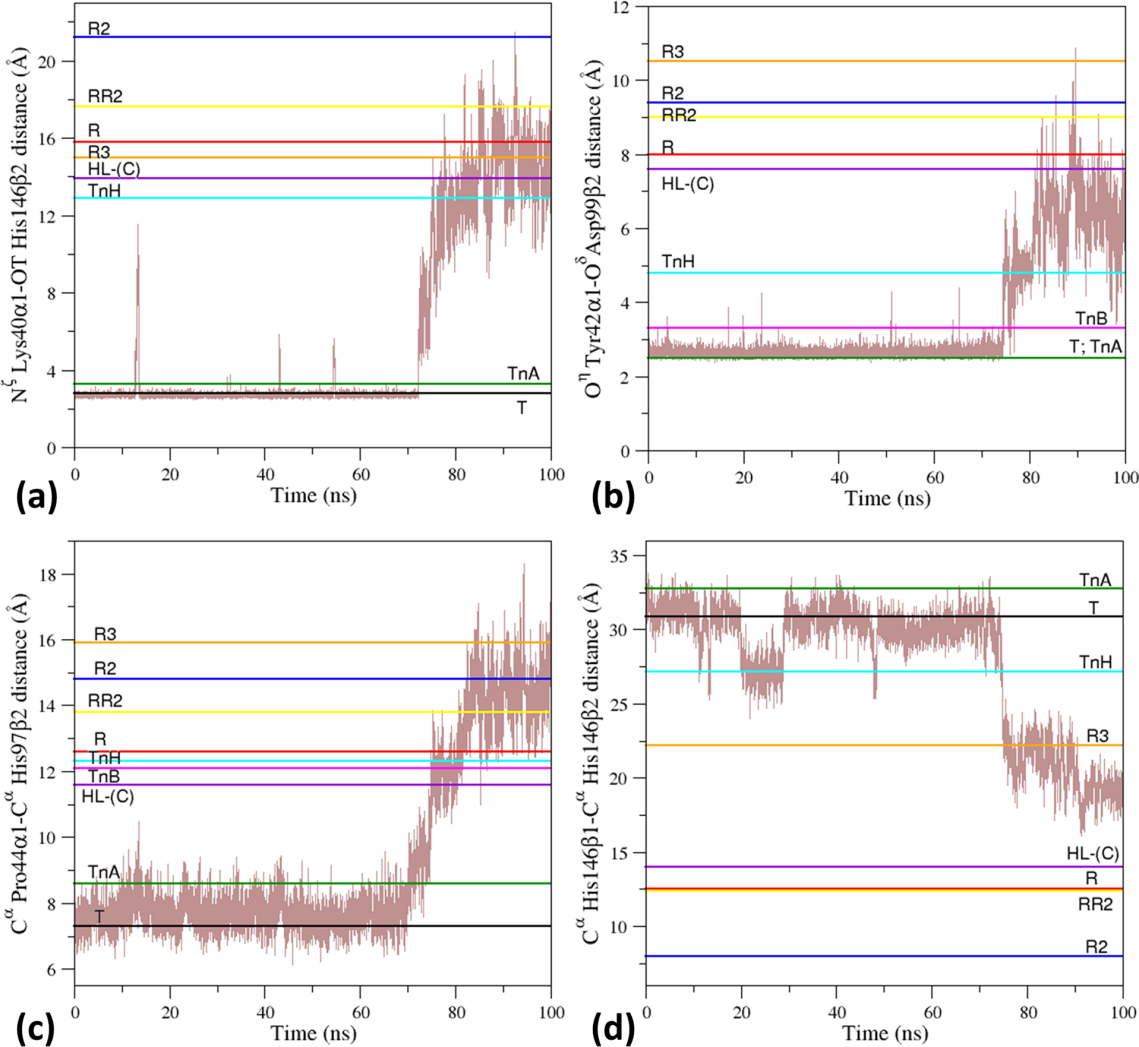
Time evolution in the T0 simulation of the structural probes that are characteristic of the different HbA states. Specifically, the distances (a) N^ζ^ Lys40α1–OT His146β2, (b) O^η^ Tyr42α1–O^δ^ Asp99β2, (c) C^α^ Pro44α1–C^α^ His97β2, and (d) C^α^ His146β1–C^α^ His146β2 are monitored.

As shown in Table S2 and Figure 4, most of these structural parameters, which present striking differences between the T- and the R-state, well discriminate these two conformational states. Moreover, the analysis of the values adopted by these parameters in the R2, RR2, and R3 states clearly indicates that they fall outside the T–R pathway. Notably, the inspection of the values of these probes in the HbA and HbTn intermediate states provides interesting insights into their location along the T–R pathway. The half-liganded state HL-(C) of HbA lies in the T–R pathway, although it is located in the proximity of the R-state (Figure 4). This observation suggests that this structure is representative of the early structural events that characterize the transition from the R- to the T-state. Consequently, the atoms involved in the interactions that strongly stabilize the T-state (O^η^ Tyr42α1–O^δ^ Asp99β2 and N^ξ^ Lys40α1–OT His146β2) are far apart in this R-like state. Interestingly, the extension of this analysis to the putative intermediate states detected in the crystallographic studies on HbTn suggests that TnA, TnB, and TnH are structurally located in the functional T–R pathway of HbA also when these local probes are analyzed (Figure 4). Moreover, Figure 4 also indicates that TnA, TnB, and TnH are located in different phases of this transition. Indeed, TnA is essentially a T-like state as the values adopted by these structural parameters do not significantly differ from those exhibited by the T form. For TnB and TnH, these probes display values that are generally rather distinct from those exhibited by both the R and the T-state. TnB assumes either T-like or R-like values for the distances O^η^ Tyr42α1–O^δ^ Asp99β2 (Figure 4b) or C^α^ Pro44α1–C^α^ His97β2 (Figure 4c), respectively. Notably, TnH appears to be essentially equidistant from the endpoints of the transition as it presents, for most of the parameters (the distances N^ζ^ Lys40α1–OT His146β2 and O^η^ Tyr42α1–O^δ^ Asp99β2) values that are in-between when compared to those exhibited by the T- and the R-state (Figure 4a,b). On the other hand, the C^α^–C^α^ distances between Pro44α1–His97β2 and His146β1-His146β2 of TnH present T-like and R-like values, respectively (Figure 4c,d).

The analysis of time evolution of these structural parameters in the T0 MD simulation indicates that all of these probes suddenly change when the overall structural T–R transition, detected by monitoring the RMSD values (Figure 2a), occurs. Indeed, the H-bonding interactions N^ζ^ Lys40α1–OT His146β2 and O^η^ Tyr42α1–O^δ^ Asp99β2 that strongly stabilize the T-state are rapidly lost at ∼ 75 ns (Figure 4a,b). Concurrently, the distance between the residues located in the switch region α1C−β2FG at the interface between the dimers α1β1 and α2β2 (C^α^ Pro44α1–C^α^ His97β2) starts to increase, adopting R-like values (Figure 4c). Accordingly, also the distance between the C^α^ atoms of the terminal His146 residues of the two β chains decreases, although the values adopted by the post-transition trajectory frames are higher than those exhibited by the crystallographic R-structure (Figure 4d).

The characterization of the intermediate states of AntHb has frequently highlighted the concomitance of the quaternary structure modifications with a significant deformation of the heme pocket.^26,29,30^ In particular, these crystallographic analyses have indicated a clear scissoring motion of the EF corner that allocates the heme prosthetic group and a swing-out movement of the distal histidine side chains that protrude from the heme pocket toward the solvent. The scissoring motion was evaluated here by considering the distance between the C^α^ atoms of the proximal His (located in the helix F) and the distal His (located in the helix E). The evolution of this C^α^–C^α^ distance indicates that no major deformation of the heme pocket occurs (Figure S7). Indeed, the values obtained are similar to those observed in canonical Hbs.^26,29,30^ The swing-out motion of the distal histidines was monitored by checking the χ_1_ and χ_2_ dihedral angles of these residues throughout the simulation. As shown in Figure S8, the distal histidines essentially assume a single rotameric state that corresponds to their canonical position within the heme pocket.

### Essential Dynamics Sampling

The HbA structural transition was also explored using a different approach based on the essential dynamics sampling (EDS). Using this methodology, we were able to explore, in addition to the T–R pathway, also the R–T transition that did not emerge from the classical MD of the R-state both in our (data not shown) and in literature simulations.^33^

**Figure 5:**
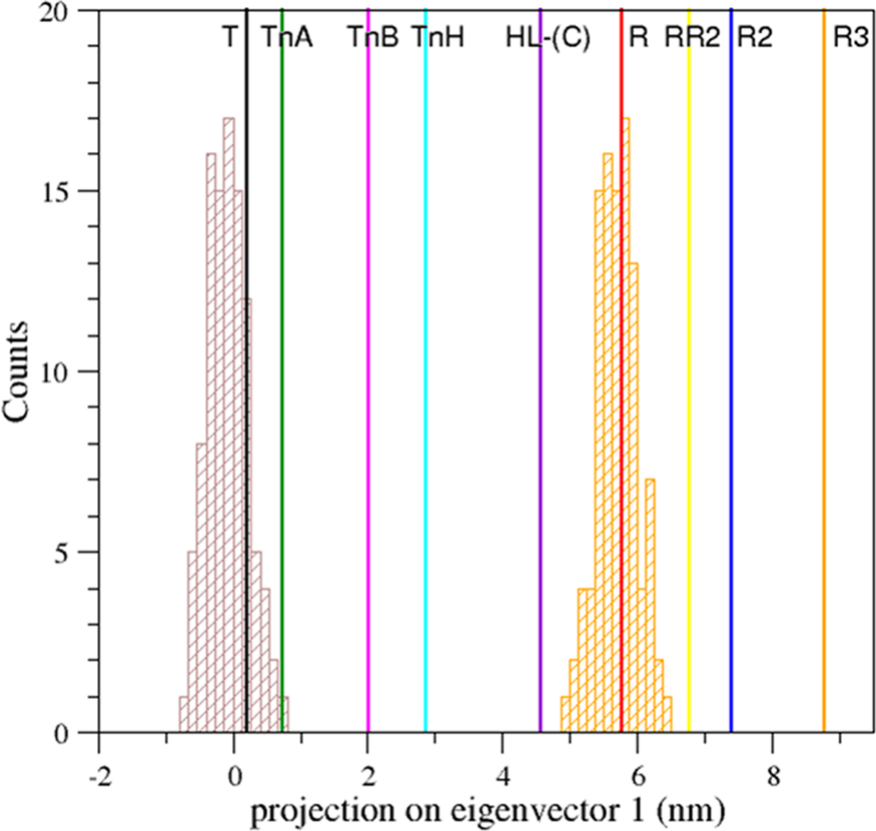
Projection on the first eigenvector of the trajectory structures extracted from the first 1 ns of the T0 (gray) and R4 (orange) simulations. The vertical lines correspond to the projections of the crystallographic structures of human HbA states—T (black, PDB ID: 2DN2), R (red, PDB ID: 2DN1), intermediate HL-(C) (violet, PDB ID: 4N7P), R2 (blue, PDB ID: 1BBB), RR2 (yellow, PDB ID: 1MKO), and R3 (orange, PDB ID: 4NI0)—and that of HbTn intermediates—TnA (green, PDB ID: 5LFG), TnB (magenta, PDB ID: 5LFG), and TnH (cyan, PDB ID: 3D1K).

**Figure 6:**
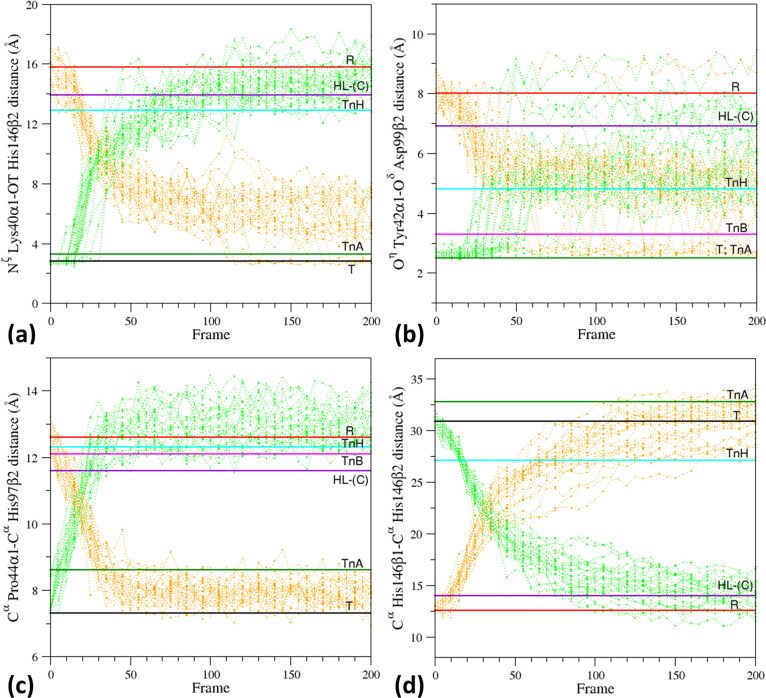
Evolution in the EDS of the structural probes that are characteristic of the different HbA states. Specifically, the distances (a) N^ζ^ Lys40α1–OT His146β2, (b) O^η^ Tyr42α1–O^δ^ Asp99β2, (c) C^α^ Pro44α1–C^α^ His97β2, and (d) C^α^ His146β1–C^α^ His146β2 are monitored in the T–R (green) and R–T (orange) trajectory structures.

EDS was applied by preliminarily generating an ensemble of T- and R-like structures necessary to extract the eigenvectors used to apply the EDS procedure. The structures present in the first ns of the T0 simulation, which present RMSDs against the crystallographic T-state in the range of 0.5–1.0 Å, constituted the basin of the T-like structures. A reliable basin of the R-state was generated by performing a short (1 ns) MD simulation, using the same protocol applied for the T0 simulation, starting from the fully ligated R-state (R4) (PDB ID: 2DN1). Also, in this case, the models of the R-like basin present RMSDs <1.0 Å when compared to the canonical crystallographic R-structure. Using the two sets of MD structures, we computed the eigenvectors that describe the overall protein transition. The movements along the eigenvector with the highest eigenvalue accounted for 82.9% of the protein total motion. We, then, projected in the space defined by the first eigenvector the ensembles of the structures corresponding to the two basins as well as the relevant structural states of HbA and HbTn considered throughout this work (Figure 5). As expected, the structures of the two basins are very close to the canonical R- and T-states. In this representation, the structures of the intermediates are located between the structures of the R and T basins, whereas the R2, RR2, and R3 fall outside this interval on the R side. This observation indicates that the first eigenvector is able to provide a reliable description of only the Hb functional transition. Upon eigenvector calculation, EDS was performed by randomly selecting five structures of the T-like basin and five structures of the R-like basin as starting points of the simulations of the T–R and the R–T pathway, respectively. As shown in Figure S9, both the T–R and the R–T transitions occur within a limited number of steps. The analysis of the plateau observed after the transition indicates that the final structures present RMSD values of ∼ 2.5 Å compared to the starting structures in line with the results obtained with the classical MD (Figure 2). The evolution of the structural probes indicates that the interactions N^ζ^ Lys40α1–OT His146β2 that stabilizes the T-state are rapidly lost in the simulation of the T-state (Figure 6a). Almost simultaneously, the interaction between O^η^ Tyr42α1 and O^δ^ Asp99β2 also is essentially lost (Figure 6b). The C^α^–C^α^ distances between Pro44α1–His97β2 and His146β1–His146β2 also assume R-like values in the post-transition structures of the T-state simulation (Figure 6c,d). Almost inverse trends are observed when these probes are monitored in the R–T EDS trajectories. In these simulations, the atoms involved in the N^ζ^ Lys40α1–OT His146β2 and O^η^ Tyr42α1–O^δ^ Asp99β2 H-bond/electrostatic interactions come rather close, although the H-bond is sporadically observed (Figure 6a,b). This is probably due to the fact that the EDS is dictated by the overall motions of the protein along the eigenvectors and not by the formation of local, specific interactions. It is worth noting that in general, the EDS structures pass through the crystallographic intermediate states and that the O^η^ Tyr42α1–O^δ^ Asp99β2 distance observed in several post-transition structures of the R-state simulations closely resembles that observed in the TnH state.

**Figure 7:**
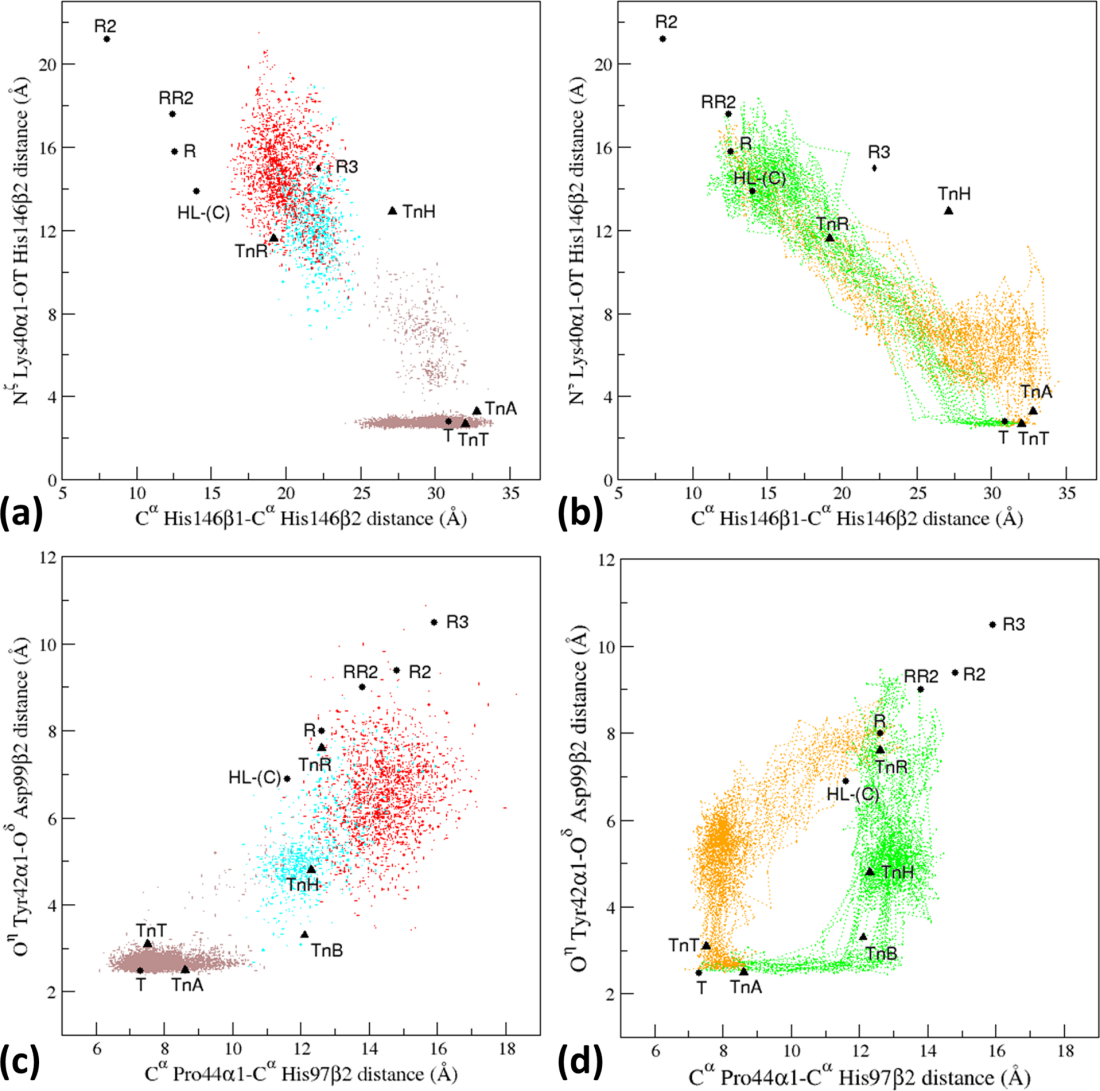
Projections of the T0 trajectory structures (a, c) and of the T–R (green) and R–T (orange) trajectory structures generated by the EDS analysis (b, d) in the space defined by some structural probes that are characteristic of the various HbA states. The points corresponding to the structures detected in the pretransition (<75 ns), transition (75–83 ns), and post-transition (>83 ns) time interval of the T0 simulation are colored in gray, cyan, and red, respectively. The representative crystallographic structures of HbA (black circles) and HbTn (black triangles) are also reported. TnB is not reported in panels (a) and (b) as the C-terminal residue of the β-chains (His146) is missing.

Finally, we monitored the coevolution of pairs of structural probes to check whether they changed independently or in a simultaneous way. From the analysis of Figure 7, it is evident that these parameters evolve in the T–R transition in a similar way in both the classical MD and the EDS. Moreover, the N^ζ^ Lys40α1–OT His146β2 distance changes concomitantly with the distance C^α^ His146β1–C^α^ His146β2 (Figure 7a,b). On the other hand, the variations of the distances between O^η^ Tyr42α1–O^δ^ Asp99β2 and C^α^ Pro44α1–C^α^ His97β2 are essentially independent. The EDS procedure, which provides information on both R–T and T–R transitions, clearly indicates that modifications at the α1CD−β2FG interaction site (C^α^ Pro44α1–C^α^ His97β2 distance) precede the break/formation of the T-state stabilizing the locking of O^η^ Tyr42α1–O^δ^ Asp99β2 interaction (Figure 7c,d). Fluctuations of the C^α^ Pro44α–C^α^ His97β2 distance are allowed in both the locked and unlocked states. In this scenario, the three intermediate states detected in the crystallographic analyses of HbTn perfectly fit into the T to R pathway. As shown in Figure 7d, the EDS simulations also indicate in the R–T transition the occurrence of a highly populated state, specific to TnH, that has not been experimentally detected yet.

## Discussion

The structural organization of tetrameric Hbs represents an admirable example of how the juxtaposition of two paralog globin chains generates an efficient system that is highly responsive to external stimuli and to specific heterotrophic factors. Very recently, using the ancestral reconstruction protein approach,^34^ it has been shown that modern Hb evolved from an ancient monomer that initially evolved by forming a noncooperative homodimer with high oxygen affinity.^35^ Intriguingly, in the ancestral progenitors of tetrameric Hb, co-operativity was acquired by reducing the oxygen affinity as a direct link between the oxygen binding site and the oligomerization interface seems to be a primordial property of this protein. Then, the appearance of the α2β2 tetramer provided a considerable enhancement of functional capabilities^36^ that made this protein crucial for the evolution of virtually all vertebrate species. The relative easiness of purifying and characterizing Hbs from different species has made these proteins prototypal systems for unraveling structure–function relationships. However, the significant complexity of these Hbs formed by two chains with different oxygen affinities that also strongly depend on the tetramer binding state and on external factors (pH and heterotrophic effectors) has made functional studies particularly difficult and the related results the subject of intense debates and controversies.^7,37−39^ A similar scenario emerges from the inspection of the structural side of the relationship. Indeed, despite the incredible amount of data accumulated over the decades, the definition of the structural bases of many issues related to either the general aspects of Hbs functionality (co-operativity and modulator of effectors) or the species-specific aspects (Bohr and Root effects) has not reached a general consensus. The difficulties related to the elucidation of the structural bases of Hb co-operativity are related to the paucity of structural information available for the intermediate states of the protein R–T functional transition. Indeed, crystallographic analyses carried out on HbA have provided a clear picture only of the endpoints of the transition with extremely limited information about the intermediate states. One of the most striking differences that emerges from a comparative analysis of the structural information of Hbs isolated from different species is related to the detection of crystallographic structures with intermediate R–T states. Somehow surprising, the characterization of Antarctic and sub-Antarctic fish Hbs, despite the close structural similarity of the R- and the T-state shared with HbA, has frequently highlighted states characterized by quaternary structures lying in the R–T pathway. Here, we report MD simulations, which were performed starting from the T-state of HbA, by focusing on the relevance of the intermediate states unraveled by the crystallographic studies of the protein functional transition.

In line with previous observations,^31,33^ the simulation of the HbA T-state indicates that the protein undergoes a spontaneous transition toward the R-state. An essential dynamics analysis of the trajectory indicates that the first eigenvalue accounts for a large portion of the overall protein mobility. The projection of the canonical T and R Hbs structures on this eigenvalue also indicates that it perfectly represents the motions associated with the T–R transition. These observations suggest that HbA possesses a limited repertoire of global motions. This is in line with the observation that strong perturbations of the heme pocket, such as oxidation and hemichrome formation, in Antarctic fish Hbs lead to quaternary structure states that fall in the T–R pathway.^26,28^ Since hemichromes are preunfolded states, it is likely that the early motions associated with the unfolding process of tetrameric Hbs follow the ones associated with the physiological functionality of the protein.

The sudden HbA T–R transition (Figure 2) explains why the crystallographic detection of HbA R–T intermediates has been so elusive despite the large amount of structural data collected for this protein. Interestingly, the projection of the HL-(C) state and of the trajectory structures on the first eigenvector (Figure 3) indicates that this state is significantly populated. This observation indicates that although close to the R-state, it represents a genuine intermediate along the trajectory. The projection of the HbTn crystallographic intermediate states onto the same eigenvector indicates that the A and B tetramers essentially fall in the conformational ensemble that characterizes the T-state. Notably, the H state is almost equidistant from the R- and the T-state of HbA. However, the low population of this state in the MD suggests that it is a high-energy structure lying in the functional pathway of human hemoglobin (Figure S5). This overall picture is corroborated by the analysis of the evolution of the structural probes that distinguish the T- and the R-state of HbA.

The general structural similarity of the HbA trajectory structures lying in the R–T trajectory with those that emerged from crystallographic studies on Antarctic fish Hbs indicates that in addition to the endpoints, these Hbs also share a similar deoxygenation pathway despite a distance of hundreds of millions of years in the evolution scale.

The HbA functional transition was also studied using the essential dynamics sampling (EDS) approach with a twofold purpose. On one side, the T–R transition, which could be seen in the classical MD, was used to validate this methodology. On the other hand, the EDS methodology was exploited to gain insights into the R–T transition that is not spontaneous in classical MD simulations. The exploration of the HbA T–R pathway by EDS highlighted its strengths and limitations compared to classical MD simulations. Although the EDS approach well reproduced the free MD trajectories, the analysis of the EDS trajectories indicates that, these simulations being driven by global motions as described by the C^α^ atoms, some local probes were occasionally not precisely reproduced. Nevertheless, by such an approach, it was feasible to monitor the reverse R–T transition that is not spontaneous in free MD simulations.^33^ The inspection of the R–T pathway indicates that it is specific to the T–R one. Indeed, the modifications at the CDα1–FGβ2 interaction site precede either the break or the formation of the T-state stabilizing interactions in the T–R and T–R transitions. On the other hand, the variations of the distances between O^η^ Tyr42α1–O^δ^ Asp99β2 and C^α^ Pro44α1–C^α^ His97β2 are essentially independent. The EDS analysis, which provides information on both R–T and T–R transitions, clearly indicates that modifications at the α1CD−β2FG interaction site (C^α^ Pro44α1–C^α^ His97β2) precede the break/formation of the T-state, stabilizing the O^η^ Tyr42α1–O^δ^ Asp99β2 interaction (Figure 7c,d). In this scenario, the three intermediate states detected in the crystallographic analyses of HbTn perfectly fit into the T to R pathway.

In conclusion, although the dynamic analysis of the HbA functional transition is a subject of long-standing investigations, the present study provides a significant contribution to this debated field as it provides a reliable three-dimensional description of the T–R HbA transition at both a global and a local level. The analogies that trajectory structures share with the intermediate states of Antarctic fish Hbs indicate that vertebrate Hbs share a similar functional pathway despite an evolutionary distance of hundreds of millions of years. Moreover, the detection of a population of states that resembles the HL-(C) structure recently reported for HbA corroborates the concept recently described by Shibayama^16^ that the HbA transition, traditionally seen as a simple two-state switch, actually occurs through population shifts among multiple quaternary states. The successful description of the T–R transition (classical MD and EDS approaches) and of the R–T transition (EDS approach) reported here paves the way for future MD studies addressing the fine HbA regulation, as the pH does in Bohr and Root effects.^40^ Finally, the atomic-level description of the intermediate states along the pathway may be also useful for the application of sophisticated approaches, like QM//MM,^41^ addressing the coupling between global HbA structural transition and oxygen binding/release.

## Materials and Methods

### Structural Models

Fully atomistic MD simulations were carried out on the T-state of HbA. In detail, the T–R transition was followed using the high-resolution crystallographic structure of the tetrameric HbA in the deoxy form (PDB ID: 2DN2) as the starting model. Several literature studies have been devoted to p*K*_a_ measurements and calculations.^42−46^ Since the main goal of this study is the atomic-level description of the T to R transition at neutral pH, Glu/Asp and Arg/Lys residues were considered negatively and positively charged, respectively. The protonation states of the histidine residues were chosen according to Zheng et al.^47^ Following indications that emerged from previous MD simulations,^31^ the terminal His146 of the β chains was deprotonated to favor the transition. The protein structural transition was also explored using the essential dynamics sampling (EDS).^48^ To this aim (see below), an additional 1 ns-long classical MD simulation on the fully ligated R-state (R4) was carried out using the high-resolution crystal structure of the oxy form of the protein (PDB ID: 2DN1). The overall and local structural features of the trajectory models were analyzed by also considering a number of canonical states (R2, R3, and RR2) and the putative T–R intermediate half-liganded HL-(C) state of HbA (PDB ID: 4N7P). Moreover, we also compared the trajectory structures with the following states identified for HbTn: T-state (TnT, PDB ID: 3NFE), R-state (TnR, PDB ID: 1T1N), tetramers A and B (TnA and TnB, PDB ID: 5LFG), and tetramer H (TnH, PDB ID: 3D1K). Details about all of these crystallographic models are reported in Table S1.

### Protocol

Fully atomistic MD simulations of the HbA T-state were performed using the GROMACS package^49^ and the CHARMM-36 all-atom force field. The protein model was solvated with water molecules (the TIP3P model was used) in a cubic box of size 150 Å. The system was neutralized with sodium and chloride counterions achieving a salt concentration of 0.15 m/L. Electrostatic interactions were treated by means of the particle-mesh Ewald (PME) method^50^ with a grid spacing of 1 Å, a relative tolerance of 10^–6^, together with a 10 Å switching for the Lennard–Jones (LJ) interactions. The LINCS algorithm was used for constraining bond lengths.^51^ The system was first energy minimized using the steepest descent (50 000 steps) and then equilibrated in two steps. In the first phase, the system was heated to 300 K temperature (increments of 10 K) for 300 ps (NVT). Equilibration of pressure at 1 atm was then conducted for 500 ps (NpT). The velocity rescaling and Parrinello–Rahman algorithms were applied for temperature and pressure control, respectively.^52,53^ Four production runs (T0, T0b, T0c, and T0d simulations) were performed at constant temperature and pressure (NpT) at 300 K and 1 atm with a time step of 2 fs. A principal component analysis was performed using the essential dynamics technique.^54^ In detail, starting from the MD simulations, we built the covariance matrix of the protein C^α^ atomic positions. The diagonalization of such a matrix allowed us to obtain a set of eigenvectors with their associated eigenvalues. These eigenvectors represent the principal protein motions used to describe the “essential” protein modes, which frequently correspond to the functional ones. This approach made it possible to represent the protein overall dynamics in a reduced essential subspace confined within the first eigenvectors defined as principal components.

The sampling method based on the essential dynamics (EDS) was applied by preliminarily generating a basin of T- and R-like models to calculate the eigenvectors used to perform the simulations. Briefly, the EDS algorithm rejects the MD step when it brings the system farther from the target and, in this case, the structure is projected on the hypersphere defined by the set of eigenvectors so that the system distance from the target is unchanged and a new MD step can be performed. On the other hand, when the MD step brings the system closer to the target, the step is accepted and a normal “free” MD move is performed. When this algorithm is applied an appropriate number of times, a MD trajectory bringing the system from the starting geometry to the target one is obtained. This approach has been successfully applied to the modeling of large protein conformational rearrangements.^48,55,56^ The ensemble of T-like structures contains the trajectory frames of the first 1 ns of the classical simulation conducted on the T-state. A reliable basin of the R-state was generated by performing a short 1 ns-long classical MD simulation starting from the fully ligated R-state (PDB ID: 2DN1), applying the same protocol used in the simulation of the T-state. Eigenvectors that describe the overall protein transition between the T and R states were then computed using these two structural ensembles. Upon eigenvector calculation, the EDS method was applied by randomly selecting five structures from both basins used as starting points in simulations of the T–R and R–T pathways and leaving the system to move along such a set of eigenvectors. Also, the EDS simulations were performed with CHARMM-36 all-atom force field and the TIP3P water model using the GROMACS package.^49^ The gmx covar and gmx anaeig tools were used to build the covariance matrix and to calculate the projection with respect to the first eigenvector.

## Supporting Information Available

RMSD profiles and projection on the first eigenvector (Figures S1–S3); spectra of the eigenvalues (Figures S4–S5); relative free energy estimations (Figure S6); structural probes (Figures S7–S8); RMSD profiles of essential dynamics (Figure S9); structural data (Table S1); and structural probes (Tables S2–S3) (PDF)

## Notes

Coordinates of the structural models used in this study were retrieved from the Protein Data Bank (https://www.rcsb.org/). MD simulations were performed using GROMACS software^49^ (version 2018.3). Structural analyses of trajectory structures were carried out using GROMACS tools^49^ and the Visual Molecular Dynamics (VMD) program.^57^ PyMOL molecular visualization system (https://sourceforge.net/projects/pymol/) and Xmgrace software version 50125 (https://plasma-gate.weizmann.ac.il/Grace/) were used to generate figures. Authors will release MD trajectories upon article publication using Zenodo repository (https://zenodo.org/).

## Abbreviations Used

Hb: hemoglobin
HbA: human hemoglobin
AntHbs: Hbs isolated from Antarctic fish
HbTn: Hb from *T. newnesi*
TnA: Hb tetramer A from *T. newnesi*
TnB: Hb tetramer B from *T. newnesi*
TnH: Hb tetramer H from *T. newnesi*
MD: molecular dynamics
EDS: essential dynamics sampling
RMSD: root-mean-square deviation

## References

[ref1] Brunori M. Hemoglobin is an honorary enzyme. Trends Biochem. Sci. 1999, 24, 158–161. 10.1016/S0968-0004(99)01380-8. 10322423

[ref2] Perutz M. F.; Wilkinson A. J.; Paoli M.; Dodson G. G. The stereochemical mechanism of the cooperative effects in hemoglobin revisited. Annu. Rev. Biophys. Biomol. Struct. 1998, 27, 1–34. 10.1146/annurev.biophys.27.1.1. 9646860

[ref3] Monod J.; Wyman J.; Changeux J. P. On the Nature of Allosteric Transitions: A Plausible Model. J. Mol. Biol. 1965, 12, 88–118. 10.1016/S0022-2836(65)80285-6. 14343300

[ref4] Bellelli A.; Brunori M. Control of Oxygen Affinity in Mammalian Hemoglobins: Implications for a System Biology Description of the Respiratory Properties of the Red Blood Cell. Curr. Protein Pept. Sci. 2020, 21, 553–572. 10.2174/1389203721666200203151414. 32013829

[ref5] Shibayama N. Allosteric transitions in hemoglobin revisited. Biochim. Biophys. Acta, Gen. Subj. 2020, 1864, 129335 10.1016/j.bbagen.2019.03.021. 30951803

[ref6] Perutz M. F. X-ray analysis of hemoglobin. Science 1963, 140, 863–869. 10.1126/science.140.3569.863. 13942632

[ref7] Ahmed M. H.; Ghatge M. S.; Safo M. K. Hemoglobin: Structure, Function and Allostery. Subcell. Biochem. 2020, 94, 345–382. 10.1007/978-3-030-41769-7_14. 32189307 PMC7370311

[ref8] El Hage K.; Hedin F.; Gupta P. K.; Meuwly M.; Karplus M. Valid molecular dynamics simulations of human hemoglobin require a surprisingly large box size. Elife 2018, 7, e35560 10.7554/eLife.35560. 29998846 PMC6042964

[ref9] Gapsys V.; de Groot B. L. On the importance of statistics in molecular simulations for thermodynamics, kinetics and simulation box size. Elife 2020, 9, e57589 10.7554/eLife.57589. 32812868 PMC7481008

[ref10] Vesper M. D.; de Groot B. L. Collective dynamics underlying allosteric transitions in hemoglobin. PLoS Comput Biol 2013, 9, e1003232 10.1371/journal.pcbi.1003232. 24068910 PMC3777908

[ref11] Perutz M. F. Stereochemistry of cooperative effects in haemoglobin. Nature 1970, 228, 726–739. 10.1038/228726a0. 5528785

[ref12] Balasco N.; Vitagliano L.; Merlino A.; Verde C.; Mazzarella L.; Vergara A. The unique structural features of carbonmonoxy hemoglobin from the sub-Antarctic fish *Eleginops maclovinus*. Sci. Rep. 2019, 9, 18987 10.1038/s41598-019-55331-3. 31831781 PMC6908587

[ref13] Tame J. R. H. What is the true structure of liganded haemoglobin?. Trends Biochem. Sci. 1999, 24, 372–377. 10.1016/S0968-0004(99)01444-9. 10500299

[ref14] Schumacher M. A.; Dixon M. M.; Kluger R.; Jones R. T.; Brennan R. G. Allosteric transition intermediates modelled by crosslinked haemoglobins. Nature 1995, 375, 84–87. 10.1038/375084a0. 7723849

[ref15] Shibayama N.; Sugiyama K.; Tame J. R.; Park S. Y. Capturing the hemoglobin allosteric transition in a single crystal form. J. Am. Chem. Soc. 2014, 136, 5097–5105. 10.1021/ja500380e. 24635037

[ref16] Shibayama N. Allosteric transitions in hemoglobin revisited. Biochim. Biophys. Acta, Gen. Subj. 2020, 1864, 129335 10.1016/j.bbagen.2019.03.021. 30951803

[ref17] Petruk A. A.; Vergara A.; Estrin D.; Merlino A. Molecular basis of the NO trans influence in quaternary T-state human hemoglobin: a computational study. FEBS Lett. 2013, 587, 2393–2398. 10.1016/j.febslet.2013.06.006. 23770098

[ref18] Merlino A.; Vergara A.; Sica F.; Aschi M.; Amadei A.; Di Nola A.; Mazzarella L. Free-energy profile for CO binding to separated chains of human and *Trematomus newnesi* hemoglobin: insights from molecular dynamics simulations and perturbed matrix method. J. Phys. Chem. B 2010, 114, 7002–7008. 10.1021/jp908525s. 20433181

[ref19] Camardella L.; Caruso C.; D’Avino R.; di Prisco G.; Rutigliano B.; Tamburrini M.; Fermi G.; Perutz M. F. Haemoglobin of the antarctic fish *Pagothenia bernacchii*. Amino acid sequence, oxygen equilibria and crystal structure of its carbonmonoxy derivative. J. Mol. Biol. 1992, 224, 449–460. 10.1016/0022-2836(92)91007-C. 1560461

[ref20] Giangiacomo L.; D’Avino R.; di Prisco G.; Chiancone E. Hemoglobin of the Antarctic fishes *Trematomus bernacchii* and *Trematomus newnesi*: structural basis for the increased stability of the liganded tetramer relative to human hemoglobin. Biochemistry 2001, 40, 3062–3068. 10.1021/bi002297j. 11258920

[ref21] D’Avino R.; Caruso C.; Tamburrini M.; Romano M.; Rutigliano B.; Polverino de Laureto P.; Camardella L.; Carratore V.; di Prisco G. Molecular characterization of the functionally distinct hemoglobins of the Antarctic fish *Trematomus newnesi*. J. Biol. Chem. 1994, 269, 9675–9681. 10.1016/S0021-9258(17)36935-1. 8144556

[ref22] Mazzarella L.; D’Avino R.; di Prisco G.; Savino C.; Vitagliano L.; Moody P. C.; Zagari A. Crystal structure of *Trematomus newnesi* haemoglobin re-opens the root effect question. J. Mol. Biol. 1999, 287, 897–906. 10.1006/jmbi.1999.2632. 10222199

[ref23] Vitagliano L.; Bonomi G.; Riccio A.; di Prisco G.; Smulevich G.; Mazzarella L. The oxidation process of Antarctic fish hemoglobins. Eur. J. Biochem. 2004, 271, 1651–1659. 10.1111/j.1432-1033.2004.04054.x. 15096204

[ref24] Mazzarella L.; Bonomi G.; Lubrano M. C.; Merlino A.; Riccio A.; Vergara A.; Vitagliano L.; Verde C.; di Prisco G. Minimal structural requirements for root effect: crystal structure of the cathodic hemoglobin isolated from the antarctic fish *Trematomus newnesi*. Proteins: Struct., Funct., Bioinf. 2006, 62, 316–321. 10.1002/prot.20709. 16299734

[ref25] Merlino A.; Vitagliano L.; Howes B. D.; Verde C.; di Prisco G.; Smulevich G.; Sica F.; Vergara A. Combined crystallographic and spectroscopic analysis of *Trematomus bernacchii* hemoglobin highlights analogies and differences in the peculiar oxidation pathway of Antarctic fish hemoglobins. Biopolymers 2009, 91, 1117–1125. 10.1002/bip.21206. 19373928

[ref26] Vitagliano L.; Vergara A.; Bonomi G.; Merlino A.; Verde C.; di Prisco G.; Howes B. D.; Smulevich G.; Mazzarella L. Spectroscopic and crystallographic characterization of a tetrameric hemoglobin oxidation reveals structural features of the functional intermediate relaxed/tense state. J. Am. Chem. Soc. 2008, 130, 10527–10535. 10.1021/ja803363p. 18642904

[ref27] Vergara A.; Franzese M.; Merlino A.; Vitagliano L.; Verde C.; di Prisco G.; Lee H. C.; Peisach J.; Mazzarella L. Structural characterization of ferric hemoglobins from three antarctic fish species of the suborder notothenioidei. Biophys. J. 2007, 93, 2822–2829. 10.1529/biophysj.107.105700. 17545238 PMC1989692

[ref28] Riccio A.; Vitagliano L.; di Prisco G.; Zagari A.; Mazzarella L. The crystal structure of a tetrameric hemoglobin in a partial hemichrome state. Proc. Natl. Acad. Sci. U.S.A. 2002, 99, 9801–9806. 10.1073/pnas.132182099. 12093902 PMC125021

[ref29] Balasco N.; Vitagliano L.; Merlino A.; Verde C.; Mazzarella L.; Vergara A. The unique structural features of carbonmonoxy hemoglobin from the sub-Antarctic fish *Eleginops maclovinus*. Sci. Rep. 2019, 9, 18987 10.1038/s41598-019-55331-3. 31831781 PMC6908587

[ref30] Vitagliano L.; Mazzarella L.; Merlino A.; Vergara A. Fine Sampling of the R-->T Quaternary-Structure Transition of a Tetrameric Hemoglobin. Chem. - Eur. J. 2017, 23, 605–613. 10.1002/chem.201603421. 27808442

[ref31] Vesper M. D.; de Groot B. L. Collective dynamics underlying allosteric transitions in hemoglobin. PLoS Comput. Biol. 2013, 9, e1003232 10.1371/journal.pcbi.1003232. 24068910 PMC3777908

[ref32] Baldwin J.; Chothia C. Haemoglobin: the structural changes related to ligand binding and its allosteric mechanism. J. Mol. Biol. 1979, 129, 175–220. 10.1016/0022-2836(79)90277-8. 39173

[ref33] Hub J. S.; Kubitzki M. B.; de Groot B. L. Spontaneous quaternary and tertiary T-R transitions of human hemoglobin in molecular dynamics simulation. PLoS Comput. Biol. 2010, 6, e1000774 10.1371/journal.pcbi.1000774. 20463873 PMC2865513

[ref34] Siddiq M. A.; Hochberg G. K.; Thornton J. W. Evolution of protein specificity: insights from ancestral protein reconstruction. Curr. Opin. Struct. Biol. 2017, 47, 113–122. 10.1016/j.sbi.2017.07.003. 28841430 PMC6141201

[ref35] Pillai A. S.; Chandler S. A.; Liu Y.; Signore A. V.; Cortez-Romero C. R.; Benesch J. L. P.; Laganowsky A.; Storz J. F.; Hochberg G. K. A.; Thornton J. W. Origin of complexity in haemoglobin evolution. Nature 2020, 581, 480–485. 10.1038/s41586-020-2292-y. 32461643 PMC8259614

[ref36] Franklin Bunn H. Regulation of Hemoglobin Function in Mammals. Am. Zool. 1980, 20, 199–211. 10.1093/icb/20.1.199.

[ref37] Tsuneshige A.; Park S.; Yonetani T. Heterotropic effectors control the hemoglobin function by interacting with its T and R states--a new view on the principle of allostery. Biophys. Chem. 2002, 98, 49–63. 10.1016/S0301-4622(02)00084-4. 12128189

[ref38] Miele A. E.; Bellelli A.; Brunori M. Hemoglobin allostery: new views on old players. J. Mol. Biol. 2013, 425, 1515–1526. 10.1016/j.jmb.2012.12.018. 23274140

[ref39] Bellelli A.; Brunori M. Hemoglobin allostery: variations on the theme. Biochim. Biophys. Acta, Bioenerg. 2011, 1807, 1262–1272. 10.1016/j.bbabio.2011.04.004. 21565157

[ref40] Brittain T. Root effect hemoglobins. J. Inorg. Biochem. 2005, 99, 120–129. 10.1016/j.jinorgbio.2004.09.025. 15598496

[ref41] Warshel A. Computer Modeling of Chemical Reactions in Enzymes and Solutions; J. Wiley & Sons, Inc., 1991.

[ref42] Czerwinski R. M.; Harris T. K.; Massiah M. A.; Mildvan A. S.; Whitman C. P. The structural basis for the perturbed pKa of the catalytic base in 4-oxalocrotonate tautomerase: kinetic and structural effects of mutations of Phe-50. Biochemistry 2001, 40, 1984–1995. 10.1021/bi0024714. 11329265

[ref43] Sham Y. Y.; Chu Z. T.; Warshel A. Consistent Calculations of pKa’s of Ionizable Residues in Proteins: Semi-microscopic and Microscopic Approaches. J. Phys. Chem. B 1997, 101, 4458–4472. 10.1021/jp963412w.

[ref44] Borštnar R.; Repic M.; Kamerlin S. C.; Vianello R.; Mavri J. Computational Study of the pKa Values of Potential Catalytic Residues in the Active Site of Monoamine Oxidase B. J. Chem. Theory Comput. 2012, 8, 3864–3870. 10.1021/ct300119u. 26593027

[ref45] Repič M.; Purg M.; Vianello R.; Mavri J. Examining electrostatic preorganization in monoamine oxidases A and B by structural comparison and pKa calculations. J. Phys. Chem. B 2014, 118, 4326–4332. 10.1021/jp500795p. 24678966

[ref46] Stivers J. T.; Abeygunawardana C.; Mildvan A. S.; Hajipour G.; Whitman C. P. 4-Oxalocrotonate tautomerase: pH dependence of catalysis and pKa values of active site residues. Biochemistry 1996, 35, 814–823. 10.1021/bi9510789. 8547261

[ref47] Zheng G.; Schaefer M.; Karplus M. Hemoglobin Bohr effects: atomic origin of the histidine residue contributions. Biochemistry 2013, 52, 8539–8555. 10.1021/bi401126z. 24224786

[ref48] Bešker N.; Amadei A.; D’Abramo M. Molecular mechanisms of activation in CDK2. J. Biomol. Struct. Dyn. 2014, 32, 1929–1935. 10.1080/07391102.2013.844080. 24125183

[ref49] Van Der Spoel D.; Lindahl E.; Hess B.; Groenhof G.; Mark A. E.; Berendsen H. J. GROMACS: fast, flexible, and free. J. Comput. Chem. 2005, 26, 1701–1718. 10.1002/jcc.20291. 16211538

[ref50] Darden T.; Perera L.; Li L.; Pedersen L. New tricks for modelers from the crystallography toolkit: the particle mesh Ewald algorithm and its use in nucleic acid simulations. Structure 1999, 7, R55–R60. 10.1016/S0969-2126(99)80033-1. 10368306

[ref51] Hess B.; Bekker H.; Berendsen H.; Fraaije J. G. E. M. LINCS: A linear constraint solver for molecular simulations. J. Comput. Chem. 1997, 18, 1463–1472. 10.1002/(SICI)1096-987X(199709)18:12<1463::AID-JCC4>3.0.CO;2-H.

[ref52] Parrinello M.; Rahman A. Polymorphic transitions in single crystals: A new molecular dynamics method. J. Appl. Phys. 1981, 52, 7182–7190. 10.1063/1.328693.

[ref53] Bussi G.; Donadio D.; Parrinello M. Canonical sampling through velocity rescaling. J. Chem. Phys. 2007, 126, 014101 10.1063/1.2408420. 17212484

[ref54] Amadei A.; Linssen A. B.; Berendsen H. J. Essential dynamics of proteins. Proteins: Struct., Funct., Genet. 1993, 17, 412–425. 10.1002/prot.340170408. 8108382

[ref55] Milanetti E.; Trandafir A. G.; Alba J.; Raimondo D.; D’Abramo M. Efficient and Accurate Modeling of Conformational Transitions in Proteins: The Case of c-Src Kinase. J. Phys. Chem. B 2018, 122, 8853–8860. 10.1021/acs.jpcb.8b07155. 30180580

[ref56] Alba J.; Milanetti E.; D’Abramo M. On the activation and deactivation pathways of the Lck kinase domain: a computational study. J. Comput.-Aided Mol. Des. 2019, 33, 597–603. 10.1007/s10822-019-00204-0. 31077013

[ref57] Humphrey W.; Dalke A.; Schulten K. VMD: visual molecular dynamics. J. Mol. Graphics 1996, 14, 33–38. 10.1016/0263-7855(96)00018-5. 8744570

