# Supplementary material for: Quaternary Structure Transitions of Human Hemoglobin: An Atomic-Level View of the Functional Intermediate States
Source: J Chem Inf Model. 2021 Aug 10;61(8):3988–99. doi: 10.1021/acs.jcim.1c00315 (PMC9473481; doi:10.1021/acs.jcim.1c00315)
Supplement: Supplementary file 1 — ci1c00315_si_001.pdf [file ci1c00315_si_001.pdf]

Quaternary Structure Transitions of Human Hemoglobin: An Atomic-Level View of the Functional Intermediate States

Nicole Balasco[a], Josephine Alba[b,1], Marco D'Abramo[b,*], and Luigi Vitagliano[a,*]

[a]Institute of Biostructures and Bioimaging, CNR, Via Mezzocannone 16, 80134 Naples, Italy.
[b]Department of Chemistry, University of Rome Sapienza, P.le A.Moro 5, 00185 Rome, Italy.

*Correspondence: luigi.vitagliano@unina.it (L.V.); marco.dabramo@uniroma1.it (M.D.).

[1]Current address: Université de Fribourg

Supporting Information

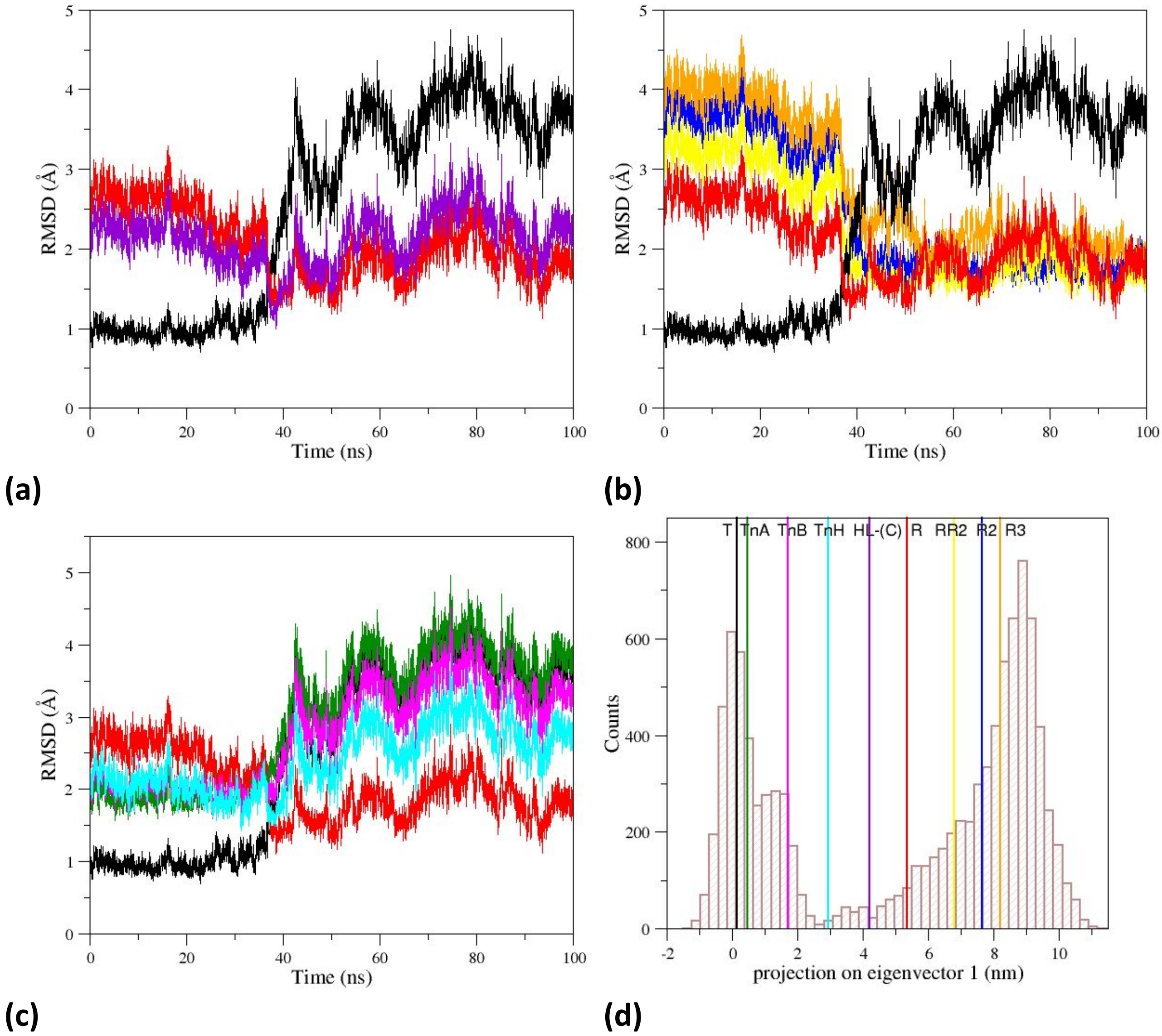

Figure S1. (a) RMSD values (computed on the C^{α} atoms) of the T0b trajectory structures versus the starting T model (black, PDB ID: 2DN2), the R state (red, PDB ID: 2DN1), and the intermediate HL-(C) state (violet, PDB ID: 4N7P). (b) RMSD values computed against the off-pathway structures R2 (blue, PDB ID: 1BBB), RR2 (yellow, PDB ID: 1MKO) and R3 (orange, PDB ID: 4NI0). (c) RMSD values computed against the intermediate states identified for HbTn: TnA (green, PDB ID: 5LFG), TnB (magenta, PDB ID: 5LFG) and TnH (cyan, PDB ID: 3D1K). RMSD values refer to the productive run without the equilibration steps producing the initial drift. (d) Projection on the first eigenvector of the T0b trajectory structures. The vertical solid lines correspond to the projections of the crystallographic structures of human HbA states: T (black, PDB ID: 2DN2), R (red, PDB ID: 2DN1), intermediate HL-(C) (violet, PDB ID: 4N7P), R2 (blue, PDB ID: 1BBB), RR2 (yellow, PDB ID: 1MKO) and R3 (orange, PDB ID: 4NI0) and of the HbTn intermediates: TnA (green, PDB ID: 5LFG), TnB (magenta, PDB ID: 5LFG) and TnH (cyan, PDB ID: 3D1K).

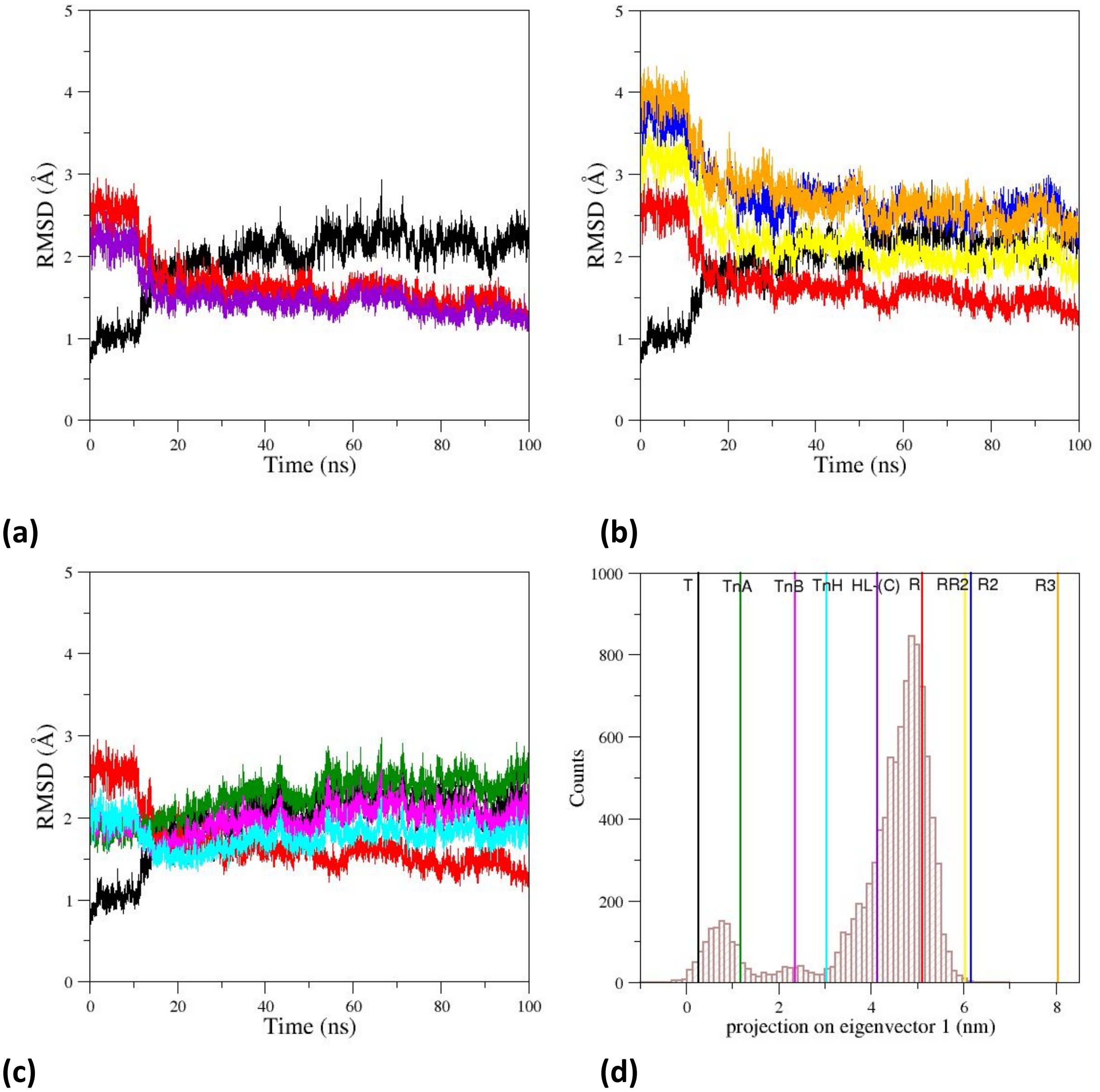

Figure S2. (a) RMSD values (computed on the C^{α} atoms) of the T0c trajectory structures versus the starting T model (black, PDB ID: 2DN2), the R state (red, PDB ID: 2DN1), and the intermediate HL-(C) state (violet, PDB ID: 4N7P). (b) RMSD values computed against the off-pathway structures R2 (blue, PDB ID: 1BBB), RR2 (yellow, PDB ID: 1MKO) and R3 (orange, PDB ID: 4NI0). (c) RMSD values computed against the intermediate states identified for HbTn: TnA (green, PDB ID: 5LFG), TnB (magenta, PDB ID: 5LFG) and TnH (cyan, PDB ID: 3D1K). RMSD values refer to the productive run without the equilibration steps producing the initial drift. (d) Projection on the first eigenvector of the T0c trajectory structures. The vertical solid lines correspond to the projections of the crystallographic structures of human HbA states: T (black, PDB ID: 2DN2), R (red, PDB ID: 2DN1), intermediate HL-(C) (violet, PDB ID: 4N7P), R2 (blue, PDB ID: 1BBB), RR2 (yellow, PDB ID: 1MKO) and R3 (orange, PDB ID: 4NI0) and of the HbTn intermediates: TnA (green, PDB ID: 5LFG), TnB (magenta, PDB ID: 5LFG) and TnH (cyan, PDB ID: 3D1K).

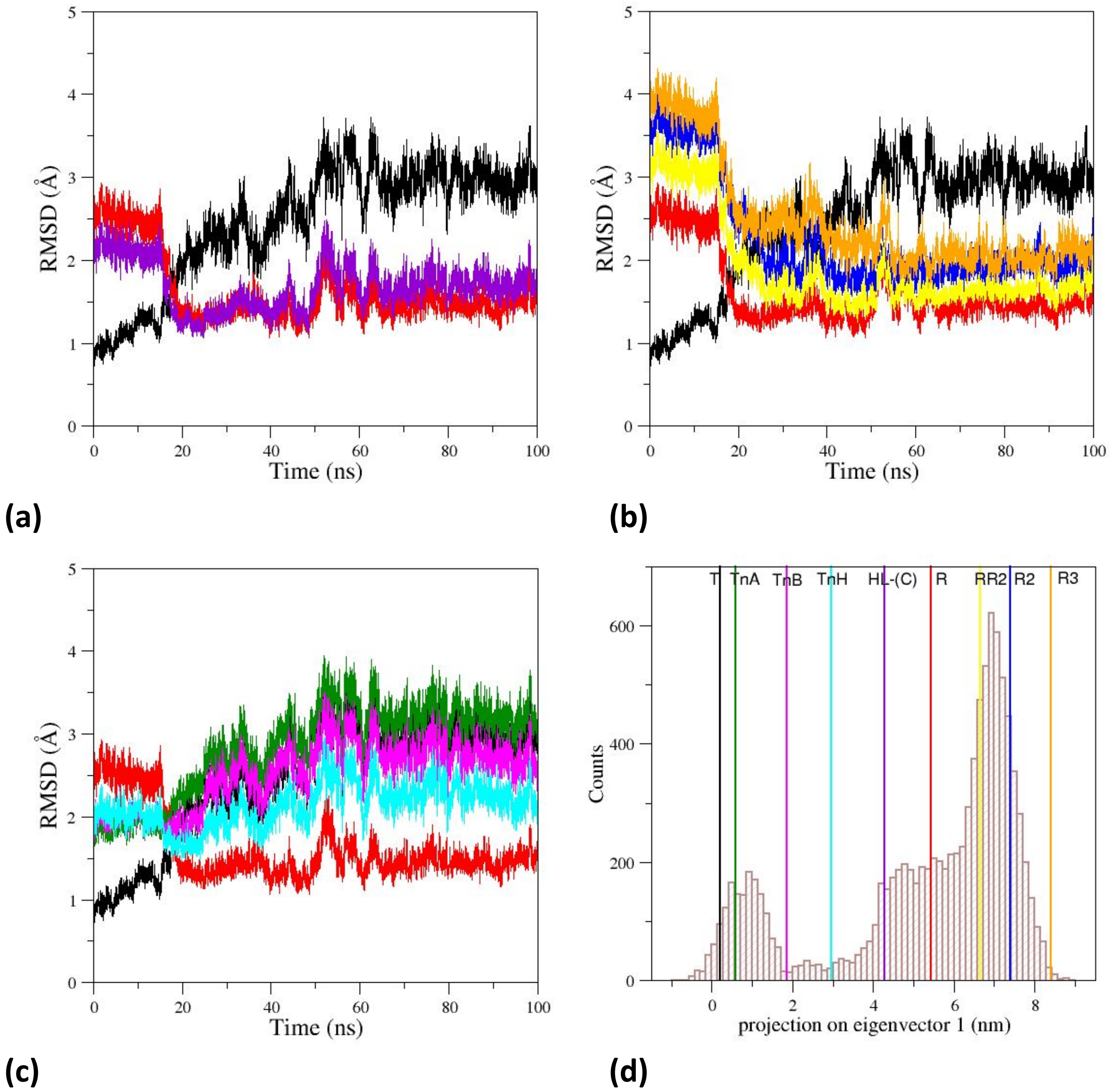

Figure S3. (a) RMSD values (computed on the C^{α} atoms) of the T0d trajectory structures versus the starting T model (black, PDB ID: 2DN2), the R state (red, PDB ID: 2DN1), and the intermediate HL-(C) state (violet, PDB ID: 4N7P). (b) RMSD values computed against the off-pathway structures R2 (blue, PDB ID: 1BBB), RR2 (yellow, PDB ID: 1MKO) and R3 (orange, PDB ID: 4NI0). (c) RMSD values computed against the intermediate states identified for HbTn: TnA (green, PDB ID: 5LFG), TnB (magenta, PDB ID: 5LFG) and TnH (cyan, PDB ID: 3D1K). RMSD values refer to the productive run without the equilibration steps producing the initial drift. (d) Projection on the first eigenvector of the T0d trajectory structures. The vertical solid lines correspond to the projections of the crystallographic structures of human HbA states: T (black, PDB ID: 2DN2), R (red, PDB ID: 2DN1), intermediate HL-(C) (violet, PDB ID: 4N7P), R2 (blue, PDB ID: 1BBB), RR2 (yellow, PDB ID: 1MKO) and R3 (orange, PDB ID: 4NI0) and of the HbTn intermediates: TnA (green, PDB ID: 5LFG), TnB (magenta, PDB ID: 5LFG) and TnH (cyan, PDB ID: 3D1K).

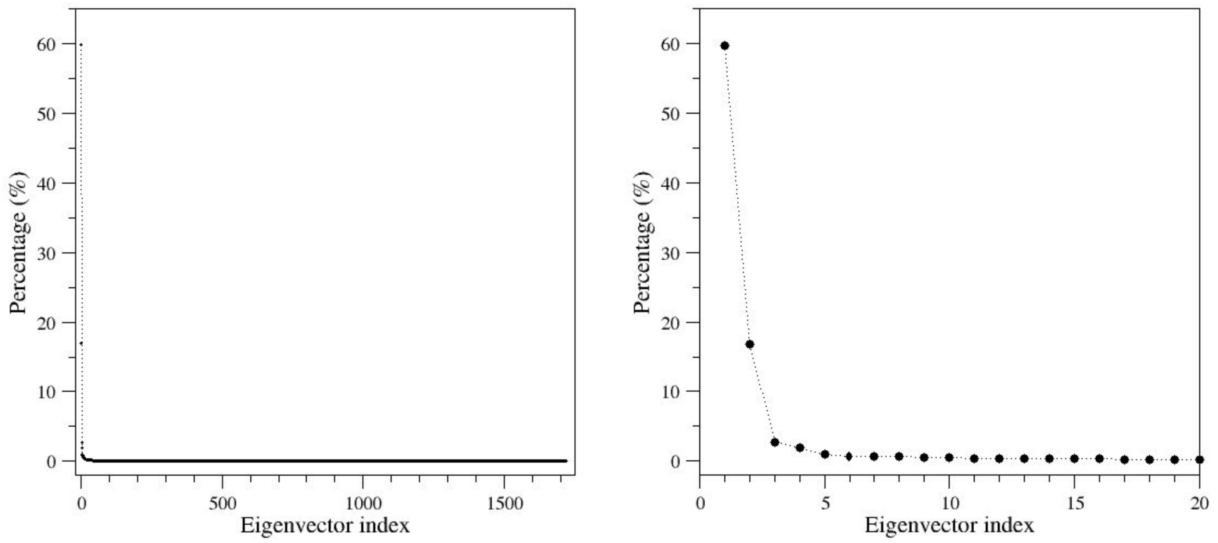

(a) **(b)**

Figure S4. (a) Spectrum of the eigenvalues (as percentage) derived from the covariance matrix diagonalization of the T0 trajectory. (b) An enlargement showing only the first 20 eigenvalues is reported.

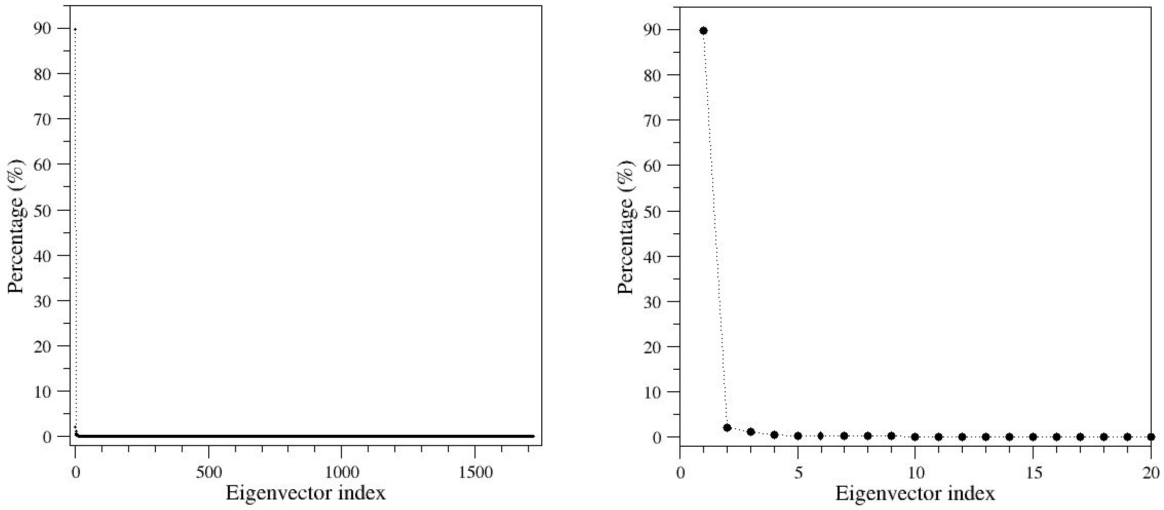

(a)

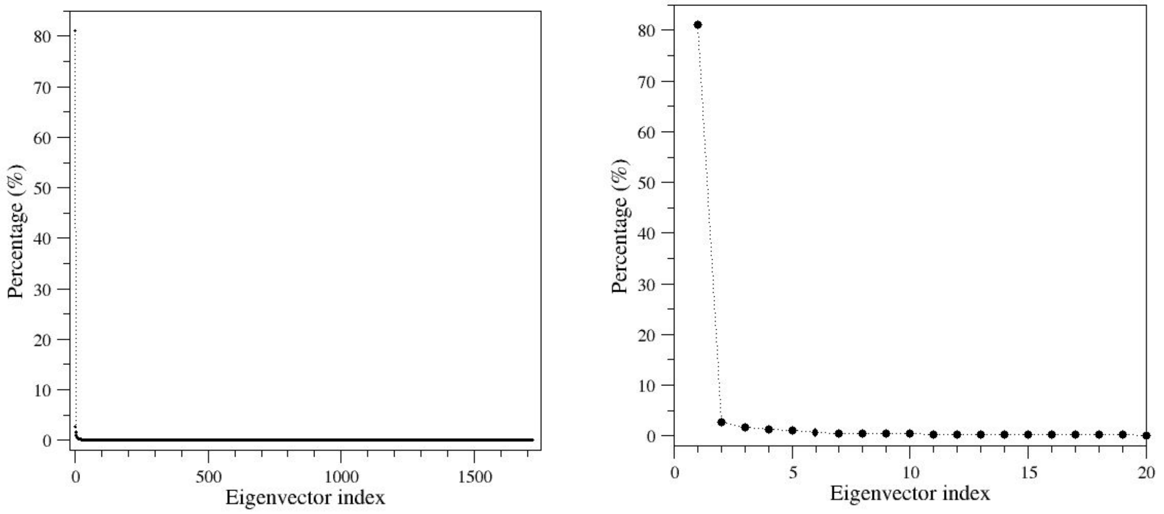

(b)

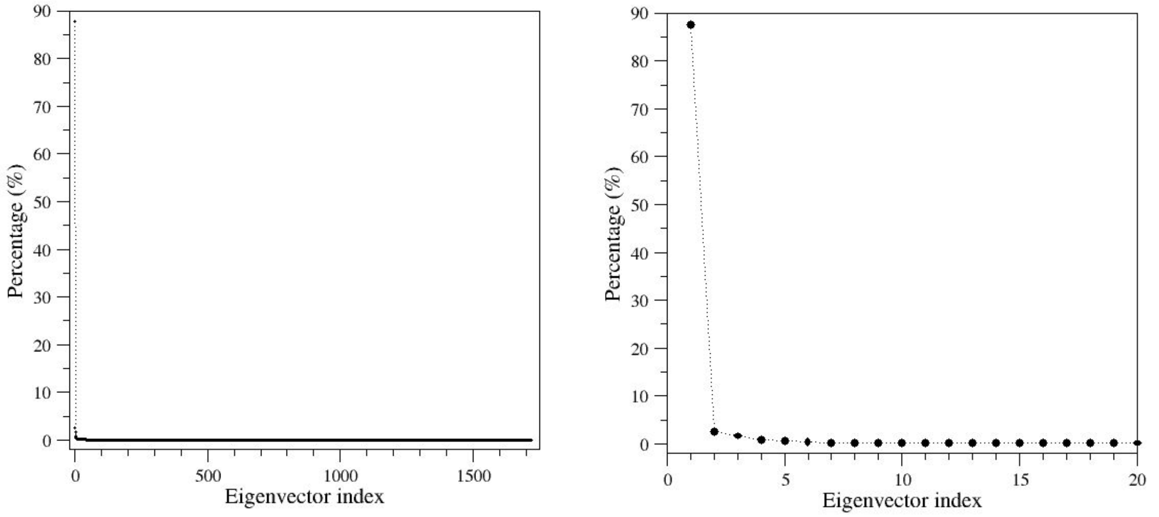

(c)

Figure S5. Spectrum of the eigenvalues (as percentage) derived from the covariance matrix diagonalization of the T0b (a), T0c (b), T0d (c) trajectories. An enlargement showing only the first 20 eigenvalues is reported on the right side of each plot.

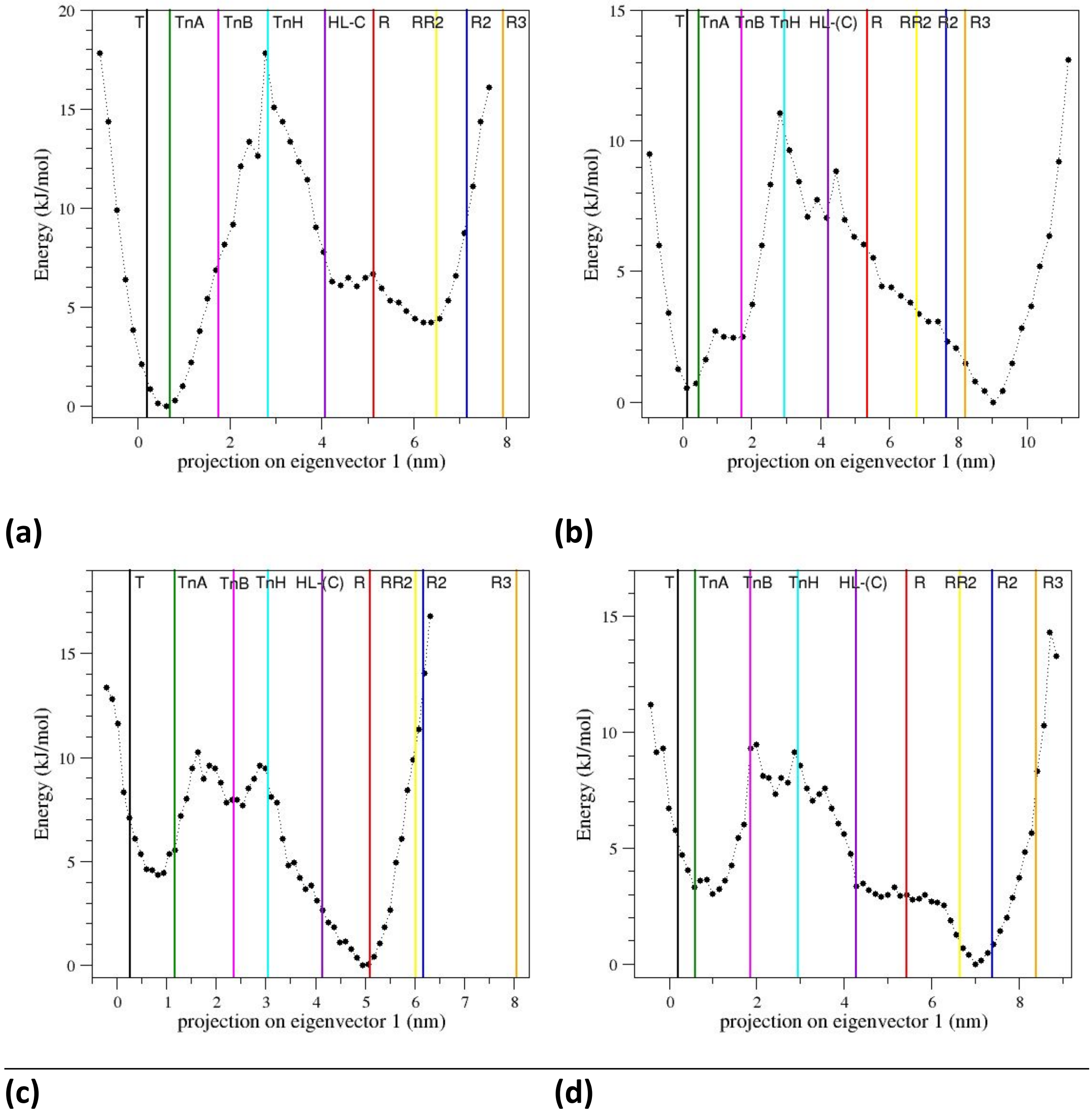

Figure S6. Estimation of the relative free energy of the different structural states detected in the T-R transition of the T0 (a), T0b (b), T0c (c), and T0d (d) MD simulations. The projection was preliminary dissected in a discrete number of states (*i*) whose populations were calculated. The black points represent the free energies as obtained by $\Delta G = -RT \ln(p_i/p_{ref})$, where p_i is the population of the structures whose projection fall in the *i* state and p_{ref} is the population of the most populated state, R is the gas constant, T is the temperature of the system, and ΔG is the free energy difference between the two states.

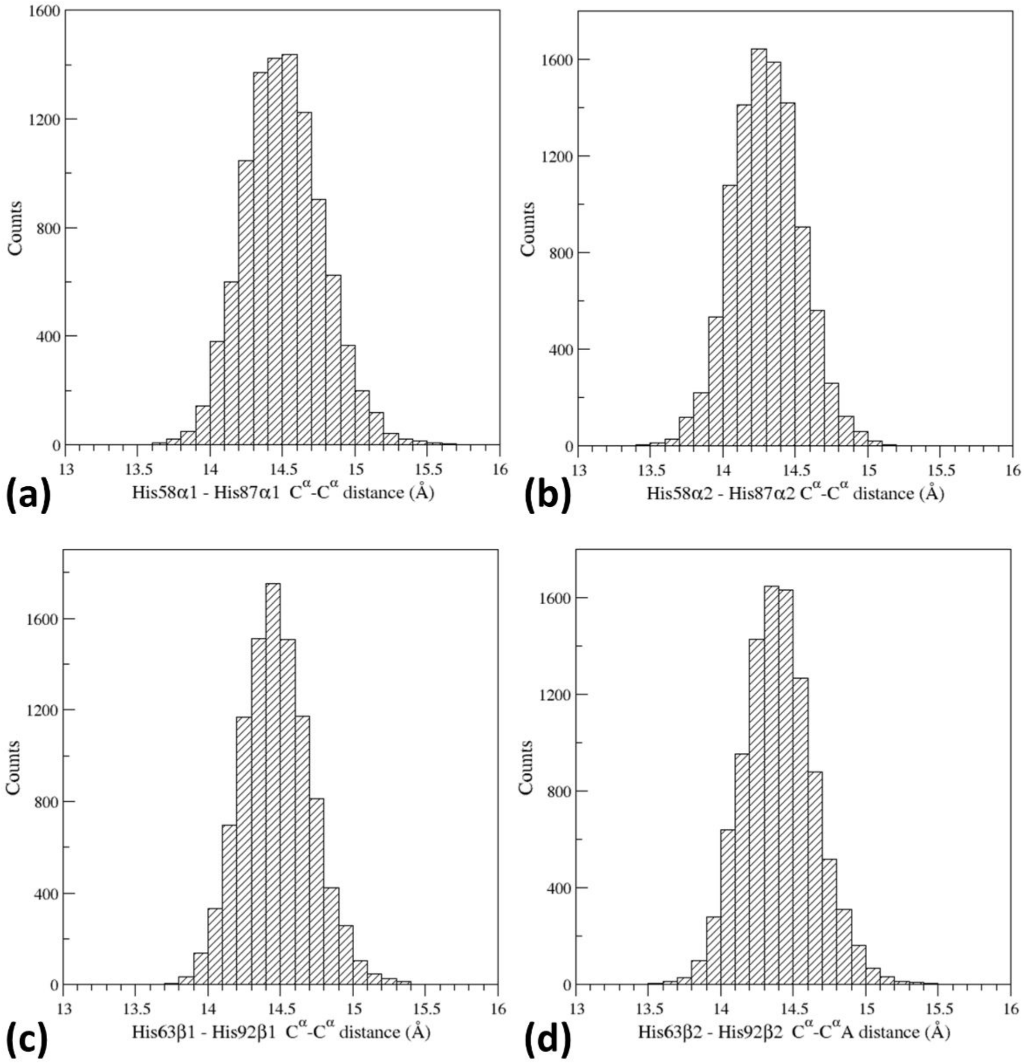

Figure S7. Distributions in the T0 trajectory structures of the distance between the C^{α} atoms of the distal and proximal His in the (a) α1, (b) α2, (c) β1 and (d) β2 chains.

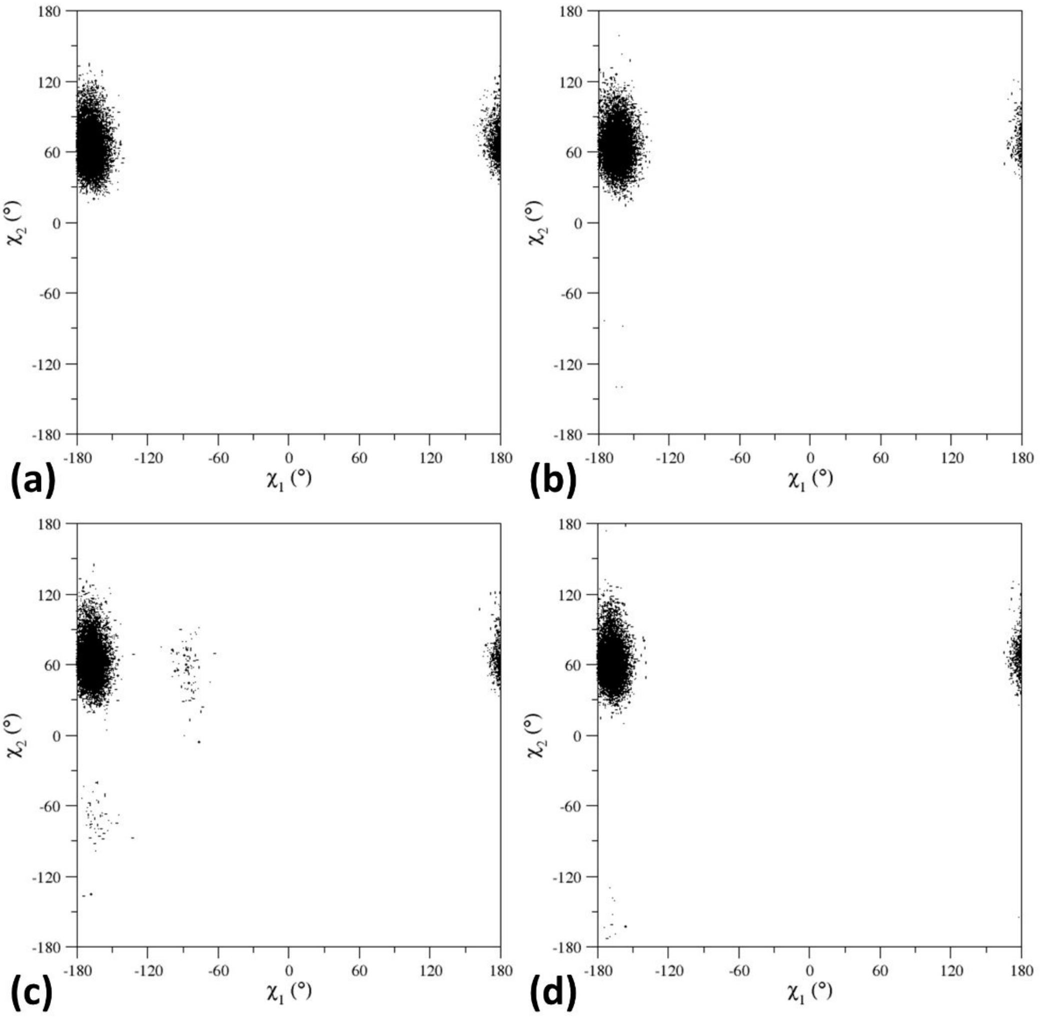

Figure S8. Distributions in the T0 trajectory structures of the χ_1 and χ_2 dihedral angle of the distal His of the (a) α1, (b) α2, (c) β1 and (d) β2 chains.

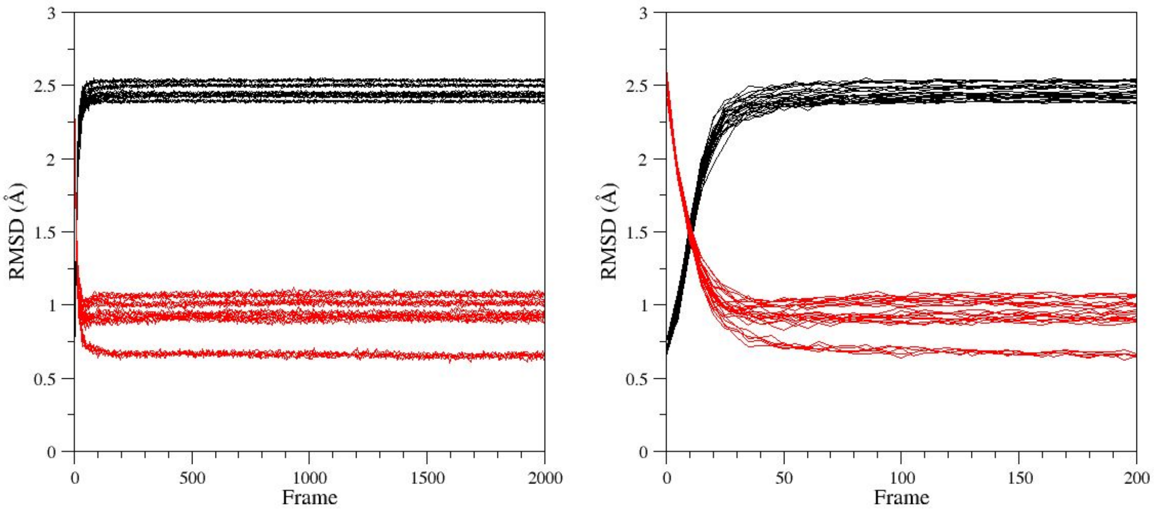

(a)

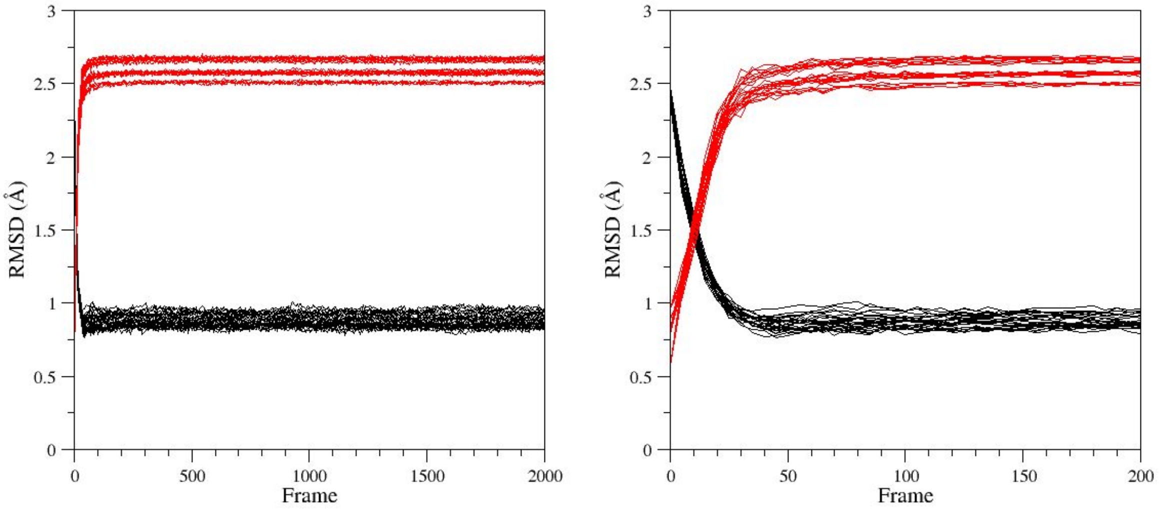

(b)

Figure S9. RMSD values, computed on the C^{α} atoms, of trajectory structures *versus* the crystallographic T (black, PDB ID: 2DN2) and R (red, PDB ID: 2DN1) states of HbA in the (a) T-R and (b) R-T EDS. A snapshot of the RMSD values including the first 200 frames is reported on the right side.

Table S1. Details of representative crystallographic structures of HbA and HbTn.

Hb State	PDB code	Resol. (Å)	R/Rfree	Asymmetric unit	Binding state
T	2DN2	1.25	0.179/null	tetramer	Unliganded
R	2DN1	1.25	0.195/null	dimer	Fully-liganded (O_2)
R2	1BBB	1.7	0.184/null	tetramer	Fully-liganded (CO)
RR2	1MKO	2.18	0.200/0.284	tetramer	Fully-liganded (CO)
R3	4NI0	2.15	0.228/0.259	dimer	Fully-liganded (CO)
HL-(C)	4N7P	2.8	0.259/0.287	3 tetramers: HL-(A), HL-(B), HL-(C)	Half-liganded (CO)
TnT	3NFE	2.01	0.185/0.250	tetramer	Unliganded
TnR	1T1N	2.20	0.192/null	dimer	Fully-liganded (CO)
TnA, TnB	5LFG	1.94	0.181/0.245	2 dimers: TnA, TnB	Fully-liganded (CO)
TnH	3D1K	1.25	0.167/0.202	dimer	Half-liganded (CO)

Table S2. Local descriptors of the structural state of representative crystallographic structures of HbA and HbTn.

Hb State	PDB code	H146β1- H146β2 C^{α}-C^{α} distance (Å)	P44α1-H97β2 C^{α}-C^{α} distance (Å)	Y42α1-D99β2 O^{η}-O^{δ} distance (Å)	K40α1-H146β2 N^{ζ}-OT distance (Å)
T	2DN2	30.9	7.3	2.5	2.8
R	2DN1	12.5	12.6	8.0	15.8
R2	1BBB	8.0	14.8	9.4	21.2
RR2	1MKO	12.4	13.8	9.0	17.6
R3	4NI0	22.2	15.9	10.5	15.0
HL-(C)	4N7P	14.0	11.6	6.9	13.9
TnT	3NFE	32.0	7.5	3.1	2.7
TnR	1T1N	19.2	12.6	7.6	11.6
TnA	5LFG	32.8	8.6	2.5	3.3
TnB	5LFG	H146 missing	12.1	3.3	H146 missing
TnH	3D1K	27.1	12.3	4.8	12.9

Table S3. Structural features of the heme pocket in representative crystallographic structures of HbA and HbTn: C^{α}-C^{α} distance between proximal and distal His residues and rotameric state of the distal His side chain.

**Hb State**	**C^{α}(HisF8)-C^{α}(HisE7) distance (Å)**				**Distal His sidechain $\chi1/\chi2$**			
	**α1**	**β1**	**α2**	**β2**	**α1**	**β1**	**α2**	**β2**
T	14.4	14.3	14.5	14.3	-171.9/61.8	-173.7/66.6	-173.0/64.3	-170.7/58.2
R	14.2	14.4	-	-	-168.8/64.4	-168.8/72.6	-	-
R2	14.5	14.5	14.4	14.5	-153.9/71.8	-164.4/78.1	-150.4/67.1	-162.5/67.2
RR2	14.1	14.4	14.1	14.2	-152.4/75.0	-174.2/80.1	-151.5/74.4	163.7/65.2
R3	14.1	14.2	-	-	-150.2/71.0	-159.0/-63.5	-	-
HL-(C)	13.8	14.3	14.1	14.4	-178.7/60.4	-157.9/55.8	-175.1/28.4	-151.1/66.5
TnT	13.4	14.3	13.1	14.1	175.1/-109.2	-164.5/64.2	174.7/-119.6	-167.4/73.5
TnR	14.3	14.3	-	-	-162.7/78.4	-154.6/90.6	-	-
TnA	14.0	13.1	-	-	-152.7/59.2	-71.9/74.9	-	-
TnB	12.8	13.1	-	-	-81.7/104.0	-71.4/80.5	-	-
TnH	14.1	14.0	-	-	-161.3/66.8	-157.7/67.9	-	-
